# Ethical Principles, Constraints, and Opportunities in Clinical Proteomics

**DOI:** 10.1016/j.mcpro.2021.100046

**Published:** 2021-01-14

**Authors:** Sebastian Porsdam Mann, Peter V. Treit, Philipp E. Geyer, Gilbert S. Omenn, Matthias Mann

**Affiliations:** 1 Department of Media, Cognition and Communication, University of Copenhagen, Copenhagen, Denmark; 2 Uehiro Center for Practical Ethics, University of Oxford, Oxford, UK; 3 Department of Proteomics and Signal Transduction, Max Planck Institute of Biochemistry, Martinsried, Germany; 4 NNF Center for Protein Research, Faculty of Health Sciences, University of Copenhagen, Copenhagen, Denmark; 5 Departments of Computational Medicine & Bioinformatics, Internal Medicine, Human Genetics, and School of Public Health, University of Michigan, Ann Arbor, Michigan, USA

**Keywords:** proteomics, systematic review, clinical proteomics, bioethics, biomedical data, incidental findings, identifiability, APOE, apolipoprotein E, GDPR, General Data Protection Regulation, IP, intellectual property, VUS, variant of unknown or uncertain significance

## Abstract

Recent advances in MS-based proteomics have vastly increased the quality and scope of biological information that can be derived from human samples. These advances have rendered current workflows increasingly applicable in biomedical and clinical contexts. As proteomics is poised to take an important role in the clinic, associated ethical responsibilities increase in tandem with impacts on the health, privacy, and well-being of individuals. We conducted and here report a systematic literature review of ethical issues in clinical proteomics. We add our perspectives from a background of bioethics, the results of our accompanying article extracting individual-sensitive results from patient samples, and the literature addressing similar issues in genomics. The spectrum of potential issues ranges from patient reidentification to incidental findings of clinical significance. The latter can be divided into actionable and unactionable findings. Some of these have the potential to be employed in discriminatory or privacy-infringing ways. However, incidental findings may also have great positive potential. A plasma proteome profile, for instance, could inform on the general health or disease status of an individual regardless of the narrow diagnostic question that prompted it. We suggest that early discussion of ethical issues in clinical proteomics can ensure that eventual health care practices and regulations reflect the considered judgment of the community and anticipate opportunities and problems that may arise as the technology matures.

## Highlights

• Principles of bioethics as they relate to clinical proteomics.
• Systematic literature review of ethics in clinical proteomics.
• Prospects for preventive proteomics profiling.
• Importance of early discussion of ethical issues to ensure eventual regulations reflect the considered judgment of the community.

## Graphical Abstract

**Figure:**
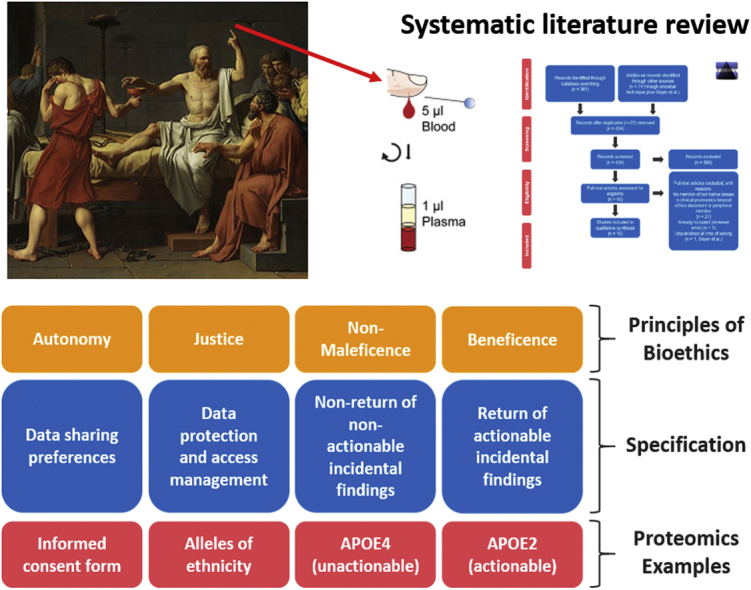

## In Brief

We introduce bioethical principles and use these as operational definitions to carry out a systematic review of the literature on ethical issues in clinical proteomics. We identify 10 ethical themes across 16 studies, many of which are familiar from other fields. We therefore survey how genomics has dealt with ethical issues and regulation. We also add our own perspectives on the ethical aspects of study design and sample treatment as well as the ethical potential of preventive proteomics profiling.

In accordance with the central dogma of molecular biology, proteins are the end product of gene expression and arguably best reflect the phenotype and functional state of an organism. Large-scale and ideally comprehensive measurements of the changes in expression levels, cellular localizations, interactions, and post-translational modifications are the subject matter of MS-based proteomics (1, 2, 3). These critical features of proteins cannot be predicted from DNA or RNA sequences. The proteome is much more complex than the inventory of less than 20,000 protein-coding genes in the human genome would suggest (4), with potentially hundreds to thousands of variant proteins forming from one gene (known as proteoforms) (5). Because of this complexity, and a variety of technological reasons, proteomics is less commonplace than workflows based on next-generation sequencing. However, over the years, the capabilities of all aspects of proteomics have vastly improved and, at the same time, a large range of specialized methods have been developed. This has made MS-based proteomics an extremely versatile tool for life scientists, allowing for explorations of simple protein expression levels as well as identification of protein–protein interactions, structural investigations, post-translational modifications, biological networks, and therapeutic targets.

As a consequence of these advances, the explanatory capacity of proteomics has increased significantly. As proteins are key biological players from a functional perspective, the ability to study the proteome in depth allows for an appraisal of the state of the entire organism. In biological contexts, this is desirable and indeed a key attraction of systems-wide technologies like proteomics. However, in a clinical context, the unbiased nature and increasing power of MS-based proteomics has increased not only the overall amount but also the proportion of particularly ethically sensitive data. Note that this only applies to untargeted proteomics, also called discovery proteomics, in which the proteome is measured to the greatest extent possible. This can be done with data-dependent acquisition or data-independent acquisition (6). Targeted proteomics methods like parallel reaction monitoring, in contrast, typically measure the levels of a handful of specific peptides (7) and are therefore conceptually more akin to existing clinical tests. Consequently, targeted proteomics does not raise the same ethical issues as system-wide proteomics does; falling back on this mode is a possible but often unsatisfactory solution. Proteomics also encompasses technologies based on antibodies (8) or other binders. Although these technologies are not explored further within this article, many of the issues discussed here are also germane to those approaches.

In an accompanying article—Geyer *et al.* (9)—we asked what kinds of information with potential ethical implications could be extracted from clinical studies with MS-based proteomics. We found that potentially personally identifiable, sensitive, and health-relevant information can be derived from a reanalysis of our previously published plasma proteomics data set on weight loss (10). Because it seems likely that information intimately related to individual persons and their health status can likewise be derived from other clinical proteomics data sets, we consider it important to broadly address associated ethical issues.

In what follows, we aim to provide a firm foundation for such an analysis, informed by our systematic review of ethical issues already identified in the clinical proteomics literature. Systematic reviews of ethical issues differ from quantitative systematic reviews in that they primarily use qualitative data (11, 12). In systematic reviews of ethical issues, articles are included where the reviewing authors judge an article to mention or discuss one or more predefined ethical issues. *Ethical* issues concern how things *should* or *ought* to be, as opposed to how things *are*. Since interpretations of ethics vary within and across cultures and political systems, we begin by introducing the conceptual background necessary to understand our operational definitions of ethical issues by relating traditional bioethical principles to some of our findings by Geyer *et al.* (9).

## The Four core Principles of Bioethics

Bioethics is a discipline that applies abstract normative principles to particular biomedical contexts. Guided by Occam's razor, we focus on the four primary principles of traditional bioethics: *nonmaleficence*, *beneficence*, *justice*, and *autonomy* (13). Each derives from millennia of debate concerning appropriate ends and acceptable means to achieving those ends. In bioethical methodology, these principles are used to specify more concrete analogs in particular biomedical contexts. For example, *nonmaleficence* appeals to the idea, at least since the Greek physician Hippocrates in the fifth Century BCE, that there is a duty not to cause harm. In the context of the incidental findings mentioned previously and in the accompanying article, this principle might be specified as a policy of *not* communicating incidental findings of uncertain significance or indicating predispositions for which no treatment currently exists. *Beneficence* refers to the ethical desirability or ideal of benefiting people, including specifically the individuals tested. In the same context, the abstract ideal of benefiting people might translate into a policy of communicating such findings if they contain, or are later found to contain, information relevant to an individual's diagnosis, prognosis, and treatment, or their general health and well-being.

The principle of *justice* concerns fairness and equality. Specifications of this principle in the research context include laws against discrimination, the practice of disclosing conflicts of interest, the need for representative databases, and generalizable and reproducible results, as well as the taboo against plagiarism. Finally, *autonomy* refers to the ideal of respecting people's choices regarding their own life and actions. When defining the term, the 18th Century philosopher Kant stressed that the capacity for rational thought enabled humans to choose laws (*nomos*) of behavior for themselves (*auto*) (14). To be both rational and consistent, Kant argued that autonomous agents must recognize the autonomy of other rational agents. Kant referred to this mutual recognition and respect as human dignity. Perhaps most importantly, he argued that dignity implied the equal worth of all autonomous agents. The concept of dignity is the intellectual foundation for international human rights law and responsible for much of the focus on equality in normative ethics. The requirement of informed consent is an example of a specification of the principle of autonomy in research contexts.

The added value of bioethical principles to discussions of ethical issues in biomedicine is essentially their usefulness as a framework for the identification and clarification of normative values at stake in a given situation. Although the principles themselves do not provide a formal equation for solving difficult issues, they do promote a discourse in which the values behind disagreements over contentious issues may be discussed with greater clarity and precision.

**Fig. 1:**
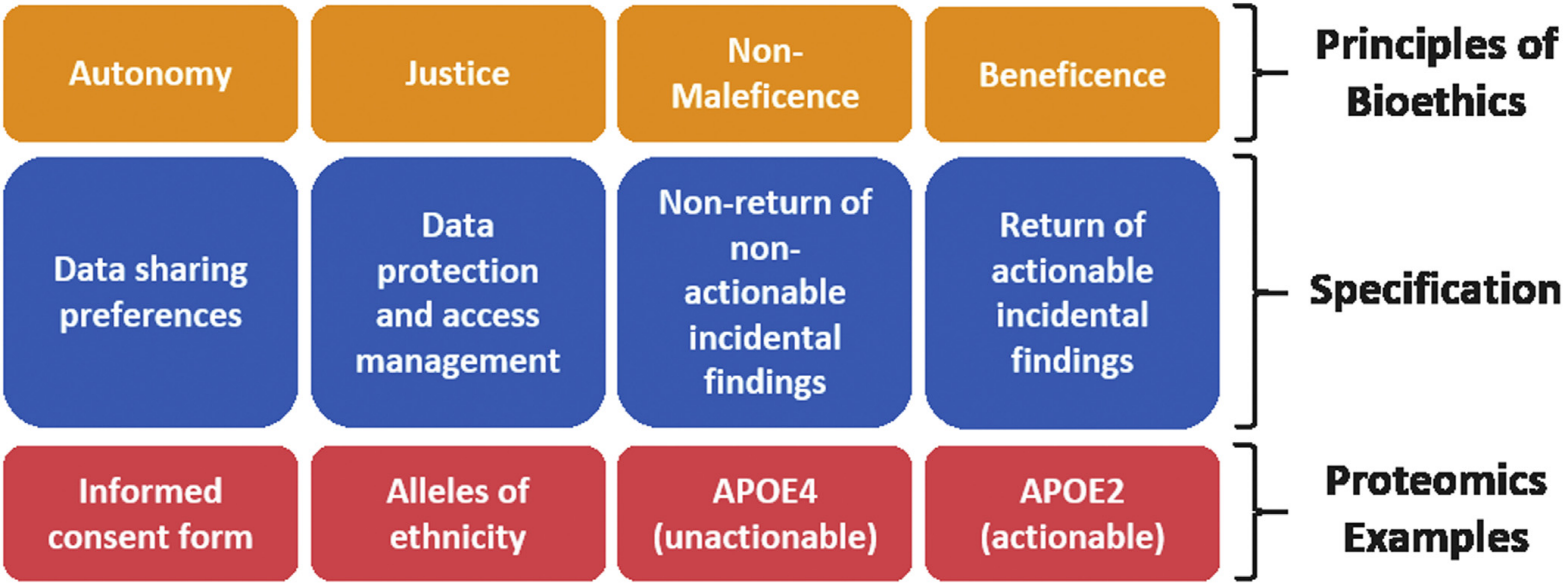
**Examples of specifications and concrete proteomic examples for the bioethical principles.** APOE, apolipoprotein E.

While these considerations may appear abstract and far removed to the proteomic researcher or clinician utilizing clinical proteomics results, they have a direct bearing as to how we should strive to guide their dissemination. Indeed, they will help determine what data should be acquired in the first place. To this end, we next illustrate these principles in concrete examples (Fig. 1).

## The Four Principles Applied By Geyer *et al.* (9)

We demonstrated that apolipoprotein E (APOE) allele status can be inferred from the plasma proteome. The presence of APOE4 alleles, which are associated with markedly increased odds of developing incurable Alzheimer's disease, is an example of a currently unactionable finding. To the extent that receiving such information might cause psychological distress, the principle of nonmaleficence would weigh against the communication of that result to the individual.

By contrast, although the presence of APOE2 alleles indicates increased risk of cardiovascular disease, this information is medically actionable since statins and lifestyle changes can be used to ameliorate the increased risk. The principle of beneficence would therefore motivate returning this information to the individual. In our reanalysis of the weight loss study, we found that the plasma proteome contains other actionable health information, including cardiovascular disease risk, a panel of proteins indicating systemic inflammation levels, and protein glycation levels indicating the presence of infection or diabetes status, respectively.

However, in both cases of actionable and unactionable health-relevant findings, the principle of autonomy requires respecting the individual's own preference as to whether to receive such incidental findings. Similarly, the return of actionable medical information can enhance the autonomy of individuals by giving them greater control over, or insight into, their health status, enabling them to act accordingly. Our reanalysis also demonstrated that samples from study participants could readily be rematched to other samples of the same participant based on individual-specific protein expression patterns or based on peptides reporting the presence of particular SNPs derived from DNA sequencing. Especially when information is individually identifiable, respect for the autonomy of individuals requires that their interest and preferences in controlling the use and storage of information concerning themselves should be taken into account.

Finally, we demonstrated that potentially sensitive information can be derived from the plasma proteome, such as biomarkers for biological sex (sex hormone–binding globulin), pregnancy (pregnancy zone protein), and ethnicity (coding SNPs that are unequally distributed across ethnicities). Where such information is obtained by unauthorized third parties, there is a risk that it might be unjustly misused for discriminatory purposes.

## Experimental Procedures

We carried out a systematic review of normative issues raised in the extant literature in relation to clinical proteomics. Drawing on the bioethical background introduced previously, we operationally defined an ethical issue for the purposes of this systematic review if (1) the issue was explicitly stated to be normative, ethical, bioethical, regulatory, or jurisprudential by the authors; or (2) the issue was discussed with direct reference or obvious implication to common bioethical principles (justice, autonomy, beneficence, and nonmaleficence), or (3) their common specifications and synonyms included informed consent, benefits, benefit sharing, fairness, equality, rights, harm, and dignity, as described previously.

**Fig. 2:**
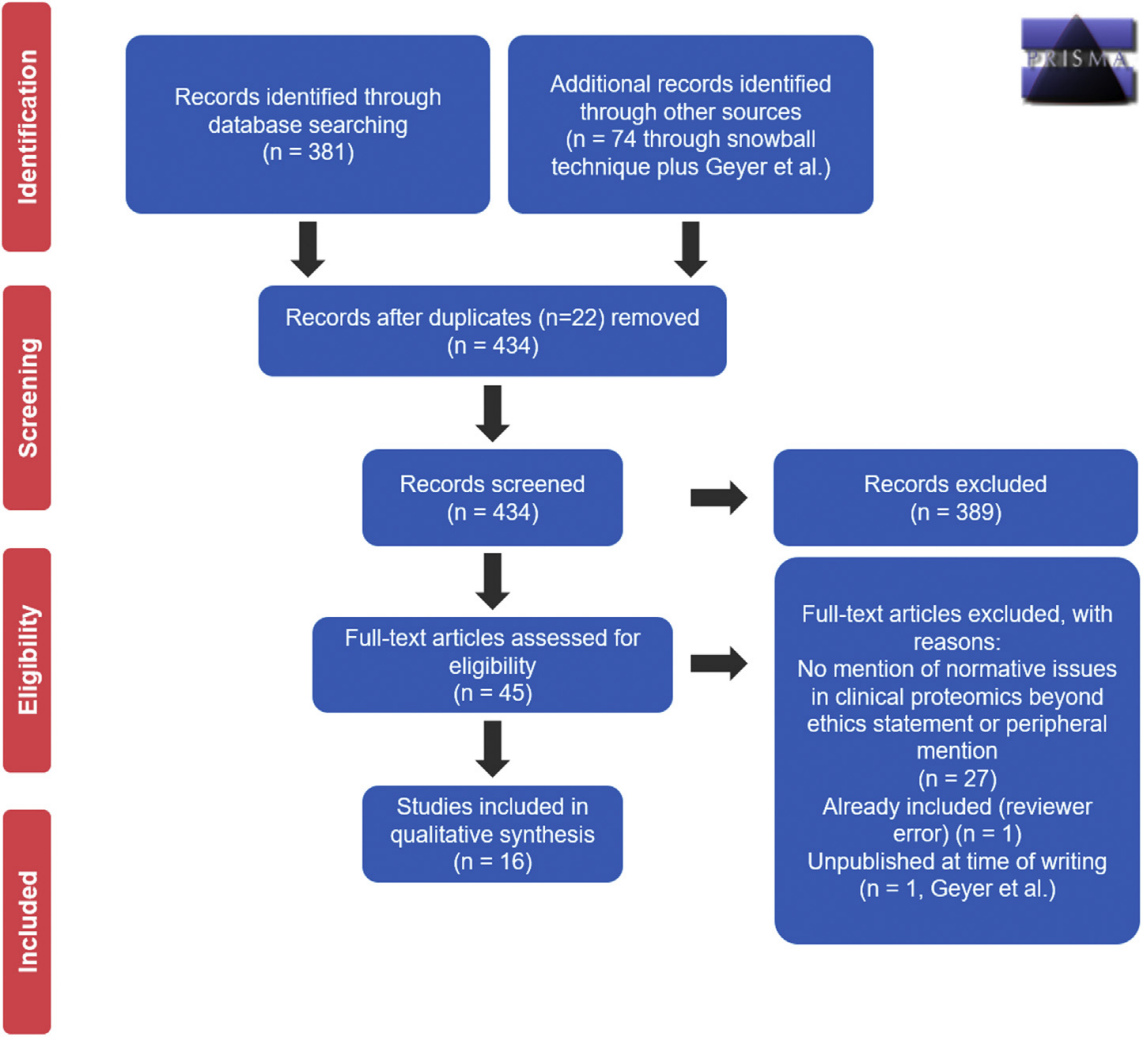
**The Preferred Reporting Items for Systematic Reviews and Meta-Analyses 2009 flow diagram.** The Preferred Reporting Items for Systematic Reviews and Meta-Analyses flowchart visualizes the flow of information through sequential phases of our systematic review (16).

We followed the ENTREQ Checklist (supplemental Table S2) (15) and the Preferred Reporting Items for Systematic reviews and Meta-Analyses flowchart (16) (Fig. 2) for reporting of systematic reviews. Systematic reviews of ethical topics within sciences are increasing, with numerous methodologies based on slight variations on traditional protocols; for an overview, see Ref. (12). Our goal was to identify any articles mentioning normative or bioethical issues in relation to clinical proteomics.

Exploratory searches using variations of the terms proteomics and ethics (proteomics∼ AND ethics∼ in Lens.org) resulted in thousands of hits, of which the vast majority contained only a simple mention of human subjects/institutional review board approval; did not differentiate between proteomics, genomics, or other omics; studied nonhuman populations; or were otherwise clearly irrelevant given our inclusion and exclusion criteria. We therefore turned to the empirical literature on search optimization, which recommended methods for maximizing the relevance of hits (17). Although the choice of search strategy may mean that we have missed some relevant studies, an expansive rather than exhaustive approach was considered reasonable given (1) the low signal-to-noise ratio and (2) our goal of identifying sufficient issues in the extant literature to generate useful qualitative themes for discussion. Using Lens.org, we searched the following databases: PMC (PubMed Central), PubMed, Microsoft Academic, Crossref, and CORE on June 22, 2020, using the following search syntax:

(mesh_term.mesh_heading:(“Proteome” OR “Proteomics”) OR field_of_study: (Proteomics OR Proteome) OR title:(clinical proteom∗) OR abstract:(clinical proteom∗) OR abstract:proteomics∼) AND (mesh_term.mesh_heading:(“Bioethical Issues”) OR mesh_term.mesh_heading:“Bioethics” OR mesh_term.mesh_heading:“Ethics” OR mesh_term.mesh_heading:(“Ethical Analysis”) OR mesh_term.mesh_heading:(“Ethics, Medical”) OR mesh_term.mesh_heading:(“Ethical Theory”) OR source.asjc_subject: (“Issues, ethics and legal aspects”) OR title:ethic∗ OR title:moral∗ OR abstract:moral∗ OR abstract:ethic∗ OR abstract:bioethic∗ OR title:bioethic∗)

This returned 381 results. Following the recommendations of the most recent systematic review on software tools for the conduct and management of systematic reviews, we uploaded these results to the systematic review Web app Rayyan for further processing (18). After removal of 16 duplicates, 365 unique articles were retained. We compared these against the following selection criteria for article inclusion:

It noted, mentioned, discussed, referred to, or highlighted one or more of the four bioethical principles or their specifications as defined previously in relation to clinical proteomics; AND

• The mention was not limited to ethical approval for the study conduct or to a peripheral mention of ethics. By way of illustration, one excluded study noted that “[t]he lack of such investigations in human[s] is probably due to ethical limitations” but did not contain any further mention or discussion of ethics (19).

In addition, an article was excluded if:

• The study did not distinguish proteomics from genomics or personalized medicine; or
• It was published in a language other than English, German, Danish, or Hungarian (the native language competencies of the authors). Only two studies were excluded because of language restrictions (both Spanish).

### Search Results

All results were screened by abstract by two of the authors (S. P. M. and P. V. T.). A first pass revealed nine “citation pearls”: “authoritative article[s], typically identified by experts, of particular relevance to the topic of inquiry that can be used to search for relevant and authoritative materials sharing common characteristics with the original pearl[s]” (20). We (S. P. M. and P. V. T.) hand searched the references of these pearls as well as their citing articles, resulting in the addition of 74 records, of which seven met inclusion criteria. With the addition of Geyer *et al.* (21), a total of 75 records were identified through means other than the search. Of these, six were duplicates, leading to a sum of 434 unique records for screening. In total, 395 records were excluded based on their abstract. The remaining 40 records were screened by S. P. M. and P. V. T. based on their full text. Disagreement between the screening authors based on study abstract occurred on five occasions. These five studies were screened on their full text by both reviewing authors, bringing the total number of records screened by full text to 45. All five were successfully resolved through discussion. For the other 40 articles reviewed on the basis of their full text, zero disagreements arose. In the end, 16 articles were retained (see supplemental Table S1 for study characteristics). This process also identified a substantial literature, summarized in Refs. (22, 23), on the forensic and bioarchaeological application of proteomics (study of biological remains in archaeological contexts). To maximize the relevance of our review to clinical proteomics, we decided to include only the first and most representative three articles, which point to the forensic and bioarchaeological potential of the human plasma or hair proteome (24, 25, 26). This led to the addition of the following exclusion clause:

• The study discussed forensic or bioarchaeological uses of proteomic profiling beyond the original three proof-of-concept studies.

Although we did not include our companion article Geyer *et al.* (9) directly in our review (as of the time of writing, it is unpublished), we refer to it where relevant throughout.

### Data Extraction and Synthesis

Mertz *et al.* (12) stressed the importance of clearly stating the *goals* and *informational units* sought in a systematic review of normative issues. The investigative goal of our review is *descriptive*. Descriptive reviews aim to identify *what* the relevant bioethical issues are. The informational units sought for the review were ethical issues/topics/dilemmas already identified or identifiable in the literature. Mertz *et al.* (11) defined this category of informational unit as an [o]verarching category for actions or situations where something has to be considered because of ethical reasons (or principles and values) or is an object of ethical research (*e.g.*, justice in regard to disabled persons; data protection when using ambient-assisted living technology; risk-benefit assessment in clinical trials; and dilemmas in triage situations).

### Issue Extraction and Thematic Analysis

We used a mixed-model approach to data extraction and thematic analysis, based on an adapted version of The Qualitative Analysis Guide of Leuven as described in Ref. (27). This involved creating a narrative summary for each included article on which descriptive data (issue) extraction was performed by a trained bioethicist (S. P. M.), in consultation with a clinical proteomic scientist (P. V. T.) according to the operational definitions and inclusion criteria described previously. These are included as supplemental data (S5–S(56)). The initial encoding and issue extraction process was kept deliberately wide and expansive, as we expected numerous variations on ethical themes to arise. The purpose of the issue extraction process was to identify enough such variations to increase the robustness of the thematic analysis carried out in the next stage of analysis. The number and encoding of issues identified by a competent colleague replicating our strategy therefore might well differ from ours; however, we expect any such variation to be within acceptable limits for qualitative review standards (11, 12, 28, 29) at the subsequent level of thematic identification.

The issue extraction and article summary process revealed substantial semantic overlap among the 40 issues identified. We therefore inductively grouped similar issues into 10 normative themes following the recommendations for thematic synthesis of qualitative research in systematic reviews described by Thomas and Harden (30), using the EPPI-Reviewer Web Beta platform for this task.

## Results

### Systematic Review

**Fig. 3:**
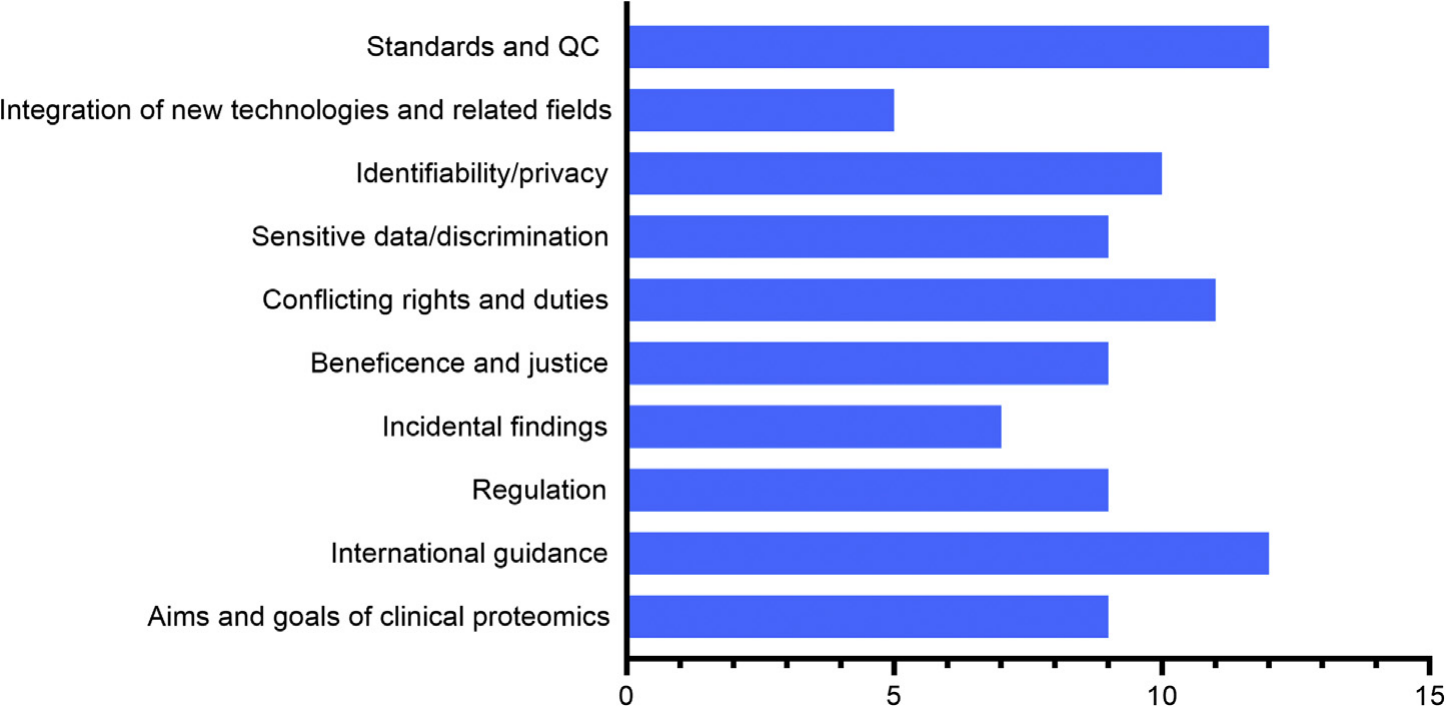
**Ten themes identified in our systematic review.** The graph visualizes the ethical themes identified by the reviewing authors (S. P. M. and P. V. T.) and the frequency with which they were identified in the 16 included articles (*x*-axis), following the methodology described in the Experimental Procedures section. Note that frequency of thematic identification is not a measure of the ethical importance of a theme. Rather, it is a descriptive visualization of the number of times these themes have been mentioned in the 16 selected articles. QC, quality control.

Ethical issues are clearly pertinent and increasingly urgent in clinical proteomics just as they are in other omics disciplines that are applied in clinical settings. As there is no systematic review of the literature in this respect, we applied rigorous methodology, combining literature database searches with terms as described in Experimental Procedures section. This included several rounds of exhaustive reading of the 16 total articles that met first and final inclusion criteria (supplemental Table S1). Figure 3 graphs the themes identified in these articles and their frequency.

#### Theme 1—Standards and Quality Control

Standards and quality control may not intuitively evoke ethical questions. However, because the clinical utility of proteomics is contingent on the quality of the underlying science, meeting appropriate standards was considered a cornerstone of ethical proteomics in many of the reviewed articles. It has been well established that an appreciation for the substantive effects of variation in all stages of the proteomic workflow renders the need to establish standard operating procedures and regular quality checks. In turn, these conventions are expected to boost reproducibility, interoperability, and cooperation among proteomics laboratories and allied fields, promoting beneficence. The stages of the proteomic workflow that were identified in the literature as ethically mandated included study design, preanalytical factors, sample collection, storage, and shipping condition (31, 32, 33). The effect of technical variation because of varied sample preparation protocols, subsequent bioinformatics analyses, and the consistent use of public databases was also acknowledged by three authors (31, 32, 34). While these concerns are being addressed by journals and granting bodies as requirements for publication or funding, respectively, our literature review adds an ethical perspective to these seemingly purely technical issues. This is readily appreciated when considering the trust and expectation of study participants, patients, and the research community at large that is implicitly bestowed on a published research project. Violating this trust by incorrect, substandard, or nonvalidated methods, when better alternatives were available, is not only a technical fault but also an ethical transgression.

#### Theme 2—Integration of New Technologies and Related Fields

The need to keep up to date with technological and scientific advances in related fields, notably other omics, to facilitate interoperability and efficiency was mentioned in five studies (32, 33, 34, 35, 36). One article proposed the use of blockchain technologies for transparent and secure data access management (32). Another pointed to the utility of connecting proteomic with the ever-increasing inflow of metadata (35).

#### Theme 3—Identifiability/Privacy

One set of concerns centers on the possibility of uniquely identifying individuals by their proteomic profile. Four early studies noted this as a hypothetical possibility for human tissue or databank studies (33, 37, 38, 39). A proof-of-concept study from 2014 demonstrated that “prominent keratin proteins served to distinguish individual profiles” from hair samples, pointing to its potential forensic use (24). It was subsequently confirmed that both individually identifying and biogeographical (*e.g.*, ethnic background) information can be derived from the hair proteome (25), and a plasma study demonstrated similar findings in the plasma proteome (26).

#### Theme 4—Sensitive Data/Discrimination

The major ethical concern regarding the use of individual-sensitive data is the possibility that information derived from them may be used to disadvantage or embarrass that individual for unfair or unnecessary reasons. A number of studies, including the one by Geyer *et al.*, clearly demonstrated this potential by showing that, for example, pregnancy, weight, ethnicity, gender, and allele status can be inferred from proteomic profiles (9, 25, 26). The potential for proteomic profiles to contain medical information that can be useful to or used against third parties, such as family members of an individual patient, was recognized by two studies (39, 40).

#### Theme 5—Conflicting Rights and Duties

Eight articles noted that duties owed to patients and/or individuals may conflict with duties owed to scientific advancement (32, 36, 37, 38, 39, 40, 41, 42). One example is where data anonymization could be employed to maximize the privacy of patients but would also create significant problems for research involving data linkage (36). Eight of the included studies noted a potential ethically relevant distinction between studies funded by or pursued for the sake of private interests *versus* publicly funded research. Finally, seven studies pointed to the possible conflicts of interest between various stakeholders in clinical proteomics, which may arise from intellectual property (IP) protections (32, 33, 36, 37, 38, 39, 42). IP protections are in place to encourage scientific and other creative progress, but current levels of protection are so high that they may also make it exceedingly difficult for scientists to access the works and materials necessary for scientific learning and advancement (33).

#### Theme 6—Beneficence and Justice

Barriers to access imposed by IP protections are especially problematic for researchers in low- and middle-income countries (33, 35, 41). Nine studies pointed to profit sharing, data sharing, or benefit sharing as ethical issues (32, 33, 35, 36, 37, 38, 39, 41, 42), especially where data silos or databases that are not representative of global human diversity present barriers to the diffusion of the benefits of scientific progress and its applications (33, 35). In addition, three studies highlighted the potential for inequitable distribution of the benefits of proteomics across or within countries because of the financial expenses and scientific expertise it requires (32, 34, 41).

#### Theme 7—Incidental Findings

Four included articles (32, 33, 36, 39) plus the article by Geyer *et al.* (9) noted the potential for incidental findings in plasma proteomics, which may be useful for medical or social purposes. Moreover, both incidental findings and findings arising from reanalysis of pre-existing data can be beneficial for advancing research (32). How such information should be stored, managed, and/or returned to individuals and databases are important questions (36), which become even more difficult in cases of findings or data of uncertain significance. The latter is such a common issue in genomics that it spawned a new term: “VUS”, “variant of unknown or uncertain significance.”

#### Theme 8—Regulation

Ten articles highlighted the importance of observing relevant national and regional regulations, especially regarding informed consent, which was often presented as a makeshift or actual solution (24, 25, 26, 31, 32, 34, 35, 36, 41, 43). However, it was noted that existing regulation may not be adequate to address issues of interoperability, efficiency, and duties owed to patients and to scientific progress; and there were calls for discussion within the field of clinical proteomics (32, 33, 35, 36, 38, 39, 41).

#### Theme 9—International Guidance

Because clinical proteomics relies on contributions from scientists all over the world and may eventually impact patient welfare on a global scale, 11 of the included articles and that of Geyer *et al.* (9) pointed to the lack of, and need for, international guidance on ethical issues (24, 31, 32, 33, 35, 36, 37, 39, 40, 41, 43), as well as the need for international collaboration on ethical and scientific issues.

#### Theme 10—Aims and Goals of Clinical Proteomics

Nine articles pointed out that such guidance should ideally go beyond regulation to address more fundamental questions, such as the goals of clinical proteomics (33, 35, 40, 41), appropriate funding priorities (33, 41), and the identification of positive ethical aims beyond the avoidance of legal liabilities (24, 32, 33, 35, 36, 37, 39, 41).

## Additional Perspectives on Bioethics and Clinical Proteomics

This concludes our literature review in a narrow sense. In the following sections, we depart from the results of our literature surveys and offer some of our own perspectives on the bioethical potential of clinical proteomics.

### Standards and Study Design

The medical potential of clinical proteomics relies on the determination of relevant differences between groups or individuals with differing health or disease states. Any inferences drawn from this technique are contingent on appropriate study design, notably sufficient statistical power to robustly indicate differentially expressed/regulated proteins (44). In plasma proteomics, the paradigm had been to analyze a very small number of samples in great depth before moving to another technology such as targeted assays that allowed for greater throughput. However, where a study is insufficiently powered to detect valuable proteins in the first stage of the biomarker discovery pipeline, all subsequent steps are doomed to failure. For these reasons, we have proposed to shift from this triangularly shaped strategy to a rectangularly shaped study design, in which several large sample sets are analyzed in parallel to increase statistical power (45).

The proteomics workflow should be evaluated to identify issues with a potential for bias. By way of illustration in a field where these factors are well studied, a recent analysis of psychological literature identified 34 researcher choices in laboratory-based study design and conduct, many of them with direct proteomic analogs, where conscious or unconscious bias may arise (46). Apart from many sources of potential issues even before sample collection, the collection phase has its own challenges, wherein the study center or scientist responsible must educate staff on best practices in preanalytical sample handling using agreed upon protocols. The importance of standardizing and controlling sample collection is pertinent in analysis of plasma, where over 50% of all published studies report proteins as follow-up candidates, which may have been introduced because of sample processing errors or as contaminants (47). Scientists are obliged by professional and normative principles to control the integrity of the proteomics pipeline by ensuring the greatest feasible quality and standardization of samples. Methods to detect bias in a study can and should be established and applied (47).

General principles of open and transparent science including sharing of methods and results and FAIR access to data (48) are especially important to fully realize the benefits of clinical proteomics in line with the ethical principle of equality and the right of everyone to enjoy the benefits of scientific progress (49, 50, 51, 52). Note, however, that these principles may conflict with the rights of autonomy and privacy of individuals—a tension that may be alleviated by technological progress in computer science such as federated learning or blockchain technologies (53, 54). In our opinion, with respect to data interpretation and analysis, the proteomics community has already come a long way by establishing widely applied guidelines such as those promulgated by the Human Proteome Organization (55, 56) and *Molecular and Cellular Proteomics* (57), requiring data sharing *via* the ProteomeXchange Consortium (58). Bioinformatics analysis and visualization of acquired data should be accessible through open-source code in order to maximize the utility and knowledge that can be derived from human proteomes. This allows other researchers to reinvestigate and reuse data sets, an essential component in replicating findings and reducing biases in study conduct and dissemination. We have previously argued that in cases where it is possible to benefit others greatly at minimal cost and effort, such as by sharing data and analyses, the ethical duty of easy rescue strongly motivates such sharing (59).

### Lessons and Challenges From Clinical Genomics

Several of the identified themes have been discussed in the ethical literature relating to clinical genomics and in the wider context of clinical and research ethics. The use of personally identifiable demographic and/or health data to discriminate against individuals is prohibited by several international instruments and national laws (60). Similarly, general privacy and health data regulations, such as the European Union General Data Protection Regulation (GDPR) and the US Health Information Portability and Accountability Act, apply to all personally identifiable and health-relevant data and are therefore relevant to clinical proteomics.

US regulations generally require informed participant consent, except for cases of research with minimal risk research where consent is impracticable or impossible to obtain. Although the GDPR imposes stricter consent requirements (consent must be specific and explicit), it also contains much broader research exemptions, including substantial public interest, preventive medicine and medical diagnosis, public health, or archiving for scientific purposes. Where data are processed without consent, appropriate safeguards, including data minimization and pseudorandomization, should be used. Processing should be proportionate to the aim pursued, carried out in accordance with the national law, and respect the “essence of the right to data privacy” (Article 9 of the GDPR). Very little guidance as to the specific meaning of these terms is included in the GDPR or available elsewhere. Very recently, Critselis (36) reported an in-depth survey of the GDPR for clinical proteomics. Under the GDPR, participant data should be readily obtainable and reviewable by participants.

The GDPR serves as the basis for similar legislation in other countries, including Israel, Brazil, and Japan (61). Although frequently not observed in practice, in principle, US regulations also provide for an individual right to access (62). National and US state laws and regulations vary and may provide greater or lower levels of protection depending on the national implementation of European Union and federal regulations (61, 63). Table 1 lists several relevant regional, international, and professional instruments (64).

**Table 1:** Regional and international regulations pertinent to clinical proteomics

Europe	United States	International
General Data Protection Regulation	Privacy Act	International Covenant on Economic, Social, and Cultural Rights
Convention on Human Rights	Health Information Portability and Accountability Act	World Medical Association Declaration of Helsinki
Convention on Human Rights and Biomedicine	Common Rule	World Medical Association Declaration of Taipei
Convention on the Protection of Individuals with Regard to the Processing of Personal Data	Affordable Care Act	Council of International Organizations of Medical Sciences International Ethical Guidelines for Health-Related Research Involving Human Subject
Charter of Fundamental Rights and Freedoms of the European Union	Clinical Laboratory Amendments Act	UNESCO Recommendation on Science and Scientific Researchers

In the discussion of return of incidental findings in genomics, the distinction between actionable and unactionable information has been considered to be of great ethical significance (65, 66, 67). Reviews of the literature generally conclude that actionable information ought to be returned to the individual or their health care provider, whereas unactionable information should not; that individuals should be informed of the likelihood of such findings at the point of consent; that individuals' preferences as expressed during the consent process regarding return of findings should be respected; and that researchers should establish an institutional review board–approved plan for the return of individual results (both individual research results and incidental findings); inform participants of this plan; clearly state the choices available to individuals; validate results prior to return; and provide for expert determination of the level of actionability of results prior to return (63, 68, 69, 70). The literature on return of incidental findings in genomics also pointed out that attempts at determining the actionability and health relevance of findings are limited by the high prevalence of VUS. There are additional issues concerning whether incidental findings should be routinely incorporated in health registries and other scientific databases as well as what should be done with incidental findings arising from reanalysis of old data (71).

In proteomics or genomics, ideally, an individual's preferences with respect to data reuse and recontact for return of incidental findings can be ascertained during the consent process. However, clearly documented informed consent is not always available for pre-existing data sources, and, in many cases, individuals may be unavailable for recontact for other reasons. In cases of uncertain significance, consent for return or storage of such information can only be partially informed. Moreover, some incidental findings may be relevant for the health of third parties, further complicating questions of consent (72). This should be much less of a concern in proteomics because dynamic protein levels rather than static genomes are measured. However, as we show in the study by Geyer *et al.* (9), sufficient genotypic information can be inferred from proteomics data to reidentify an individual based on SNPs; this raises the question of revealing carrier status in next of kin from routine proteomics profiling.

The experience of clinical genomics also foreshadows issues of justice that may become relevant to clinical proteomics. For example, it has been pointed out that polygenic risk scores are much more accurate for individuals of European descent than any other ethnicity because an estimated 79% of reference genomes describe Caucasian ancestral lines, despite these representing only 16% of the human population (73). Although we are not aware of definitive data, most reference proteomes appear to originate from Caucasians as well. Creating demographically representative databases is not only an issue of justice. It has also been shown that non-Caucasian samples contribute more associations to a Genome-Wide Association Study data set than Caucasian samples at equivalent sample sizes (74). The relatively lower levels of scientific infrastructure, funding, and access to data in low-to-middle income countries, as well as among economically disadvantaged communities in high-income countries, only exacerbate this problem. We anticipate that issues of costs and medical insurance will initially complicate access to the benefits of clinical proteomics, raising issues of distributive justice. However, the technology is developing rapidly, and we expect that costs will decrease as they have for genomic services.

Legal and bioethical scholars working in clinical genomics have warned against confusion stemming from multiple overlapping regulatory structures (75). Like clinical genomics, clinical applications of proteomics aim both at assisting the diagnosis and treatment of individual patients as well as contributing to biomedical knowledge generation. Similarly, both genomics and proteomics may be applied for public health purposes and/or in commercial contexts. This overlap is important because different ethical frameworks and legal rules apply to the clinical, research, public health, and commercial domains. As a general legal rule, in situations of doubt or conflict, the standard most protective of individual rights should be followed (75).

As is the case in clinical genomics, it is likely that health-related proteomic applications will eventually be offered both by public and private providers. Much of clinical proteomics research is publicly funded and has significant potential public value. The knowledge generated by clinical proteomics research can therefore to some extent be characterized as a public resource, which should be managed in ways that maximize public benefits (71). This does not mean that publicly supported scientists must sacrifice their scientific freedom or basic research by aiming their efforts at preconceived notions of public good to the detriment of the advancement of science. It does, however, imply that, where possible, publicly supported research should address conditions that cause widespread mortality and morbidity, should include traditionally underrepresented groups in databases and trials, and should make research results widely accessible according to the principles of open science (76). In privately funded research or commercial applications, these considerations do not apply to the same extent because the primary duties of private companies are owed to their shareholders rather than the general public. Nevertheless, it is important that this difference not be used as an excuse to exploit individuals or provide substandard services. Commercial applications of genetic tests have been criticized for failing to provide transparency and sufficient information in relation to the secondary processing, selling or sharing, and privacy and security of sensitive and health-related customer data (77). Since the future development of clinical proteomics relies on the willingness of individuals to share their data, maintaining the public's support for and trust in both research and clinical applications of proteomics is of crucial importance (78). Moreover, leading corporations are developing a long-term orientation in which not just shareholders, but employees, customers, communities, and externalities, represent major stakeholders (79).

### Proteomic Preventive Profiling

Part of the clinical promise of proteomics is its ability to capture phenotypic information. Genotypes are largely static, whereas phenotypes fluctuate according to endogenous and environmental perturbations. To make the most of this information, researchers and patients may opt to have proteomic profiles taken periodically or regularly, akin to the annual physical with a primary health care provider. Although the full realization of this approach still lies well in the future, it has significant potential to advance biomedical aims if bioethical principles can be respected.

Regular proteomic profiling has the potential to contribute to medical beneficence by providing information about environmental and endogenous influences on health, which are not easily discernible from other methods, especially if supplemented by direct measures of environmental and dietary exposures. This information could then be used to provide actionable health information to individuals and to advance biomedical understanding.

Profiles obtained from a single droplet of blood contain information relevant to multiple diagnostic and treatment purposes, potentially rendering the use of several current tests redundant. Besides potential efficiency gains, ease and convenience of access to medical services are known to correlate with better health outcomes (80). Since ethnic minorities and the poor face greater logistical obstacles in accessing health care (81), reducing the number of clinical visits necessary for testing purposes has the potential to reduce inequality in access to health care, contributing to both beneficence and justice.

Reviews of genomic research participants' perspectives have found significant interest in the return of health-relevant findings, especially where these are actionable (65, 82). Proteomic profiling could facilitate autonomy by respecting individuals' informed decisions concerning return of results and by granting individuals greater insight into, and control over, their health.

The bioethical framework of facilitating beneficence and justice while respecting the rights and autonomy of individuals can be valuable in anticipating future opportunities and problems. For example, a simple method of avoiding many ethical questions surrounding the return of incidental findings is simply not to look for them or not to analyze those parts of the proteomic profile known to relate to common incidental findings. However, in refusing to analyze or return information, which might be used to improve the health or well-being of individual participants, opportunities to benefit these individuals are missed. Obviating a set of ethical questions may seem like a good way to avoid making mistakes, but if this occurs at the cost of missed opportunities to benefit others greatly at little cost while respecting their rights, the social price paid may be too high.

Another example is the potential application of proteomic profiling to prevention and general wellness beyond the diagnosis and treatment of disease. There is no obvious morally significant reason why only those who visit their clinician for unrelated reasons should benefit from medically actionable information obtainable from a proteomic profile, especially where this information is derived from a method that might equally be applied to healthy individuals. Medical doctors are bound by history and codes of professional ethics dating to antiquity to focus primarily on the treatment of disease; prevention did not appear in modern versions of the Hippocratic Oath until 1964 in US medical schools (83). Clinical proteomics, unfettered by history, is free to define for itself a broader vision of contributing to flourishing beyond the treatment of disease. It could therefore benefit from the modern understanding of several historically neglected factors governing health and flourishing, including lifestyle, sociocultural, and environmental determinants of health (84).

Shifting from a sole focus on the diagnosis and treatment of recognized disease early in the clinical application of proteomics may help us discover and make use of opportunities to prevent diseases from manifesting in the first place. Such a shift in perspective beyond the narrow diagnostic question under consideration could potentially open additional opportunities for the general improvement of human health and welfare. The potential benefits of such an approach have been illustrated in a recent study of scientific wellness, in which multiomic, including proteomic, dense dynamic personal data clouds were profiled to identify putative biomarkers and provide targeted actionable health advice, leading to improvements in measured clinical biomarkers among participants (85).

## Conclusion

As clinical proteomics matures and affects, directly and indirectly, the lives of a growing number of people, familiar responsibilities and duties grow stronger while new ones enter the picture. The necessity for guidance and regulations governing, for example, data privacy and the sharing of important information in other medical and omics contexts is widely appreciated. However, in proteomics, these issues have lingered in the background as the field has focused on developing its foundations. In the present article and in the article by Geyer *et al.* (9), we have shown how these issues affect clinical proteomics in similar and different ways, given its especially dynamic and systemic nature.

Based on our systematic review of the relevant literature, we identified, summarized, and discussed the nascent debate on ethics in applications of clinical proteomics. Although the number of studies touching on the topic is limited, we identified 10 ethical themes across 16 included studies. We also briefly surveyed how these topics have been treated in the relevant genomics and general clinical and research ethics literature. Finally, we added our own perspectives on bioethics in clinical proteomics.

Experience from related fields shows that ethical and regulatory standards can, and eventually *will*, be imposed from outside the profession (*e.g.*, in the form of bans, regulations, and other legislation) or from within (self-regulation). We suggest that clinical proteomics should, as far as possible, aim for responsible self-regulation. This is not only because clinical proteomics scientists and collaborating physicians understand the scientific and technical context better than others. It is also because self-regulation, based on values chosen by the profession itself, ideally informed by patient advocates, has a greater likelihood of legitimacy and therefore of effect. The genomics world has learned that it is important to engage patients and patient advocates and their organizations, not just rely on health professionals to determine what is presumed to be best for the patients (86). Moreover, the experience of genomics demonstrates that professional discussions of ethical issues in clinical proteomics can benefit from the perspectives of social scientists, lawyers, ethicists, and humanists (87). We believe the gap in the extant literature represents an opportunity for participation by our community. The moment to begin exercising control over the rules and regulations that will bind us tomorrow is *soon*. The time to begin thinking and talking about them is *now*.

## Data Availability

All data is submitted in the supplementary material.

## Conflict of interest

The authors declare no competing interests.
