# Supplementary material for: Ethical Principles, Constraints, and Opportunities in Clinical Proteomics
Source: Mol Cell Proteomics. 2021 Jan 14;20:100046. doi: 10.1016/j.mcpro.2021.100046 (PMC7950205; doi:10.1016/j.mcpro.2021.100046)
Supplement: Supplemental Tables [file mmc1.pdf]

Supplemental Data For "**Ethical Principles, Constraints and Opportunities in Clinical Proteomics**"

*T1. Study characteristics:*

| Study | Year | Country | Author background | Published in | Primary focus | Reference nr. |
|---|---|---|---|---|---|---|
| Reymond et al. | 2003 | Germany | Medical (Surgery) | *Proteomics* | Normative: Sample storage and tissue banking | 37 |
| Beck | 2004 | Germany | Medical (Pathology) | *Pathology Research and Practice* | Normative: Ethical/IRB review in relation to samples | 39 |
| Nestler et al. | 2004 | Germany | Medical (Surgery) | *Expert review of proteomics* | Normative: Sample storage and tissue banking | 36 |
| Liska | 2004 | Germany | Science (Biology) | *Proteomics* | Normative: Choosing worthwhile problems to study | 40 |
| Merrill and Mazza | 2006 | USA | Science (Multidisciplinary) | *National Academies Press* | Normative: IP constraints vs progress of proteomics | 41 |
| Jackson and Davis | 2010 | UK | Science (Proteomics) | *Proteomics Clinical Applications* | Empirical: Importance of logistical challenges and variations in clinical sample storage | 30 |
| Twyman | 2012 | UK | Science (Biology) | *Encyclopedia of Applied Ethics (2nd ed., Academic Press)* | Normative: Several ethical issues discussed | 38 |
| Gupta et al. | 2014 | India | Science (Proteomics) | *Biochimica et Biophysica Acta* | Normative: Several ethical issues discussed with a special focus on low-and-middle income countries | 32 |
| Laatsch et al. | 2014 | USA | Science (Forensics) | *PeerJ* | Empirical: Proof-of-concept for individual variation in human hair proteome profiling | 23 |
| Özdemir et al. | 2015 | Turkey | Science (Multidisciplinary) | *Journal of Neural Transmission* | Normative: Analysis of several sociological factors of high relevance to clinical proteomics | 34 |
| Holmes et al. | 2016 | Canada | Social Science (Interdisciplinary) | *New Genetics and Society* | Normative: Socioanthropological comparison of the manipulatory and explanatory functions of proteomics and genomics | 43 |
| Li et al. | 2016 | USA | Data Science (Bioinformatics) | *AMIA Summits on Translational Science Proceedings* | Empirical: Demonstration of derivation of individually identifiable information from blood plasma | 25 |
| Parker et al. | 2016 | USA | Science (Toxicology) | *PLOS ONE* | Empirical: Demonstration of derivation of individually identifiable and sensitive (ethnicity) information from hair shaft | 22 |
| Martens and Vizcaíno | 2017 | Belgium/UK | Science (Proteomics and Bioinformatics) | *Trends in Biochemical Sciences* | Empirical: Overview, description, potential, and challenges of data sharing in proteomic libraries | 33 |
| Critselis | 2019 | Greece | Science (Epidemiology) | *Proteomics Clinical Applications* | Normative: Implications of the EU GDPR for clinical proteomics | 35 |
| Boonen et al. | 2019 | Belgium | Science (Proteomics and Bioinformatics), Philosophy | *Genes* | Normative: Several ethical issues discussed | 31 |

*T2. The 40 normative issues identified, grouped by frequency of mention:*

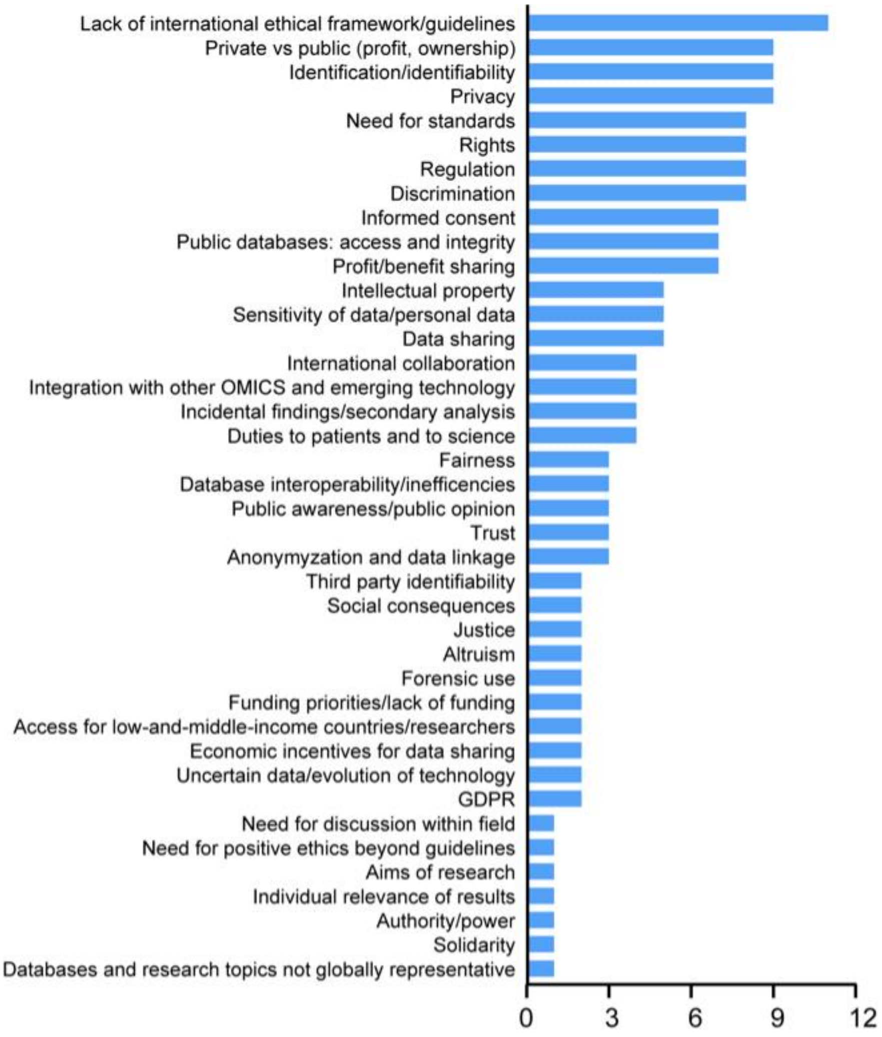


*T3. ENTREQ Checklist:*

| No | Item | Guide and description |
|---|---|---|
| **1** | Aim | What normative issues in clinical proteomics are identified or identifiable in the extant literature? |
| **2** | Synthesis methodology | Thematic synthesis |
| **3** | Approach to searching | We searched PMC, PubMed, Microsoft Academic, Crossref, and CORE on June 22nd, 2020, using a sophisticated, pre-defined search strategy (see Methods) |
| **4** | Inclusion criteria | Inclusion: It noted, mentioned, discussed, referred to, or highlighted one or more of the four<br>bioethical principles or their specifications as defined above in relation to clinical proteomics;<br>AND<br>The mention was not limited to ethical approval for the study conduct or to a peripheral mention of ethics.<br>Exclusion: The study did not distinguish proteomics from genomics or personalized medicine; OR<br>It was published in a language other than English, German, Danish or Hungarian (the native language competencies of the authors) |
| **5** | Data sources | See 3. These were the largest and most relevant collections available to the searching authors |
| **6** | Electronic Search strategy | Using Lens.org, we queried the databases mentioned in 3 using the following syntax: (mesh_term.mesh_heading:("Proteome" OR "Proteomics") OR field_of_study: (Proteomics OR Proteome) OR title:(clinical proteom*) OR abstract:(clinical proteom*) OR abstract:proteomics~) AND (mesh_term.mesh_heading:("Bioethical Issues") OR mesh_term.mesh_heading:"Bioethics" OR mesh_term.mesh_heading:"Ethics" OR mesh_term.mesh_heading:("Ethical Analysis") OR mesh_term.mesh_heading:("Ethics, Medical") OR mesh_term.mesh_heading:("Ethical Theory") OR source.asjc_subject: ("Issues, ethics and legal aspects") OR title:ethic* OR title:moral* OR abstract:moral* OR abstract:ethic* OR abstract:bioethic* OR title:bioethic*) |
| **7** | Study screening methods | All hits screened by abstract by the lead and third author. Forty-five candidates screened by full text by the lead and third author. |
| **8** | Study characteristics | See T1 above. |
| **9** | Study selection results | See PRISMA Flowchart in Experimental Methods, p. 9. |

| | | |
|---|---|---|
| **10** | Rationale for appraisal | Existence or otherwise of ethical issues as operationally defined in the methods section. |
| **11** | Appraisal items | Presence or otherwise of ethical issues as operationally defined in the methods section. |
| **12** | Appraisal process | First round of appraisal conducted by first author. Second round conducted by third author. |
| **13** | Appraisal results | We identified several issues, which we grouped into ten normative themes. |
| **14** | Data extraction | The text of the manuscripts themselves provided the raw data. Whenever a normative issue as operationally identified was discovered, it was coded using the software described below in 15. |
| **15** | Software | EPPI-Reviewer Web Beta<br>Rayyan<br>EndNote X9 |
| **16** | Number of reviewers | Two; lead and third author. |
| **17** | Coding | See 14. |
| **18** | Study comparison | The data extracted from the included studies were aggregated by ethical issue. The main unit of comparison was the existence or otherwise of one or more such issues. |
| **19** | Derivation of themes | Inductive |
| **20** | Quotations | Limitations of space did not allow for use of quotations in the main text. Instead, we extract and include the abstract and a narrative synthesis of each article in the rest of the supplementary data below and present illustrating quotes at the thematic level here.<br><br>Sensitive data/discrimination: “The study of the proteome raises a number of potential ethical issues, such as those concerning the ownership, storage, and use of human tissues; the storage and use of data arising from proteomic research (especially if this affects donor privacy or could lead to discrimination) …” (38).<br><br>Identifiability/privacy: “Our results suggested the presence of significant privacy risks in raw clinical proteomic data” (25).<br><br>Incidental findings/reuse: “… incidental findings has been a hot topic in the debate regarding genomic data and the question will be even more pertinent in proteomics.” (31). |

| | | |
|---|---|---|
| | | Standards/QC: “Clinical proteomic study and translational work therefore are highly dependent on the availability of sufficient numbers of samples, together with relevant clinical data, … a high degree of expertise is required, along with dedicated funding, personnel, data storage capacity and space, and ethical and legal frameworks to ensure the samples remain intact, accessible and relevant for future research (“future-proofing”)” (30).<br><br>Conflicting rights and duties: “The report recommends 13 actions that policy-makers, courts, universities, and health and patent officials should take to prevent the increasingly complex web of IP protections from getting in the way of potential breakthroughs in genomic and proteomic research” (41).<br><br>Need for normative international guidelines: “We would like to emphasize that it will most likely not be possible to have one answer that would fit all situations (and study conditions) but an internationally recognized set of ethical guidelines to follow up would be a step forward. This would potentially assist the ethical committees advising personalized medicine and clinical proteomics studies” (31).<br><br>Aims and goals of clinical proteomics: “The answer to the question ‘toward what goals should we apply our technological and scientific abilities?’ essentially depends on an understanding of ethics and the morals that guide our actions, whether this question is ultimately answered by a funding organization or an individual academic researcher” (40).<br><br>Benefits/Justice: “ … considerable amount of financial supports have been devoted for the notable biobanking activities … very few of them are located in or store samples from developing countries. Moreover, most of these biobanks focus on one particular kind of disease or population due to which the diverse causes of mortality that exist in developing countries are neglected” (32).<br><br>Regulation: “The challenge to HUPO is to recognize ethically significant problems needing to be addressed, determine effective experimental approaches, advise the community on these possibilities, and aid researchers in securing funding for these prospective applications” (40).<br><br>Integration of new technologies and related fields: “Recently blockchain technology is being proposed [51,52] as a distributed electronic ledger for hosting health information” (31). |
| **21** | Synthesis output | See “results” section, pp. 10-14 |

# EPPI-Reviewer output

N.B. This is the ‘report’ output of the EPPI-Reviewer Beta Web platform which we used to conduct thematic analysis. It consists of a title, abstract, narrative summary (ranging from short to long) and a tree of themes with issues grouped underneath them. We wrote these narrative summaries following the methodological recommendations cited in the methods section. This is all supplementary material and the major part of data extraction. Each small bullet point below (filled) is a theme and each round (unfilled) bullet is an issue grouped under that theme. The quotes below often include methodological solutions to the proposed issues.

ID 48757562: Beck (2004)

Beck Norbert. 2004. "Proteomics in pathology, research and practice: ethical considerations". Pathology - Research and Practice 200(2):179-180.
Abstract: A researcher should always seek advice from his responsible ethics committee. Usually, advisory discussions with physicians doing research are not problematic in ethical terms when they want to examine biological material obtained, especially, when the material is no longer needed, the material will be used anonymized, no individualizing genes will be examined, the aims of research are not disputed in ethical terms, research is not expected to yield results of individual relevance to the person affected, there is no indication that the person affected will object to research and the expenditure for obtaining the individual consent is excessively high.

Narrative summary: Points out a number of general issues related to human samples. Only distinction made is that proteomics may involve smaller quantities of material than other study methodologies and therefore may involve fewer ethical issues.

Reviewer: Sebastian Porsdam Mann, Peter Vilmos Treit
Themes
- Identifiability/privacy
  - Third party identifiability
- Incidental findings/reuse
  - Individual relevance of results
- Standards and quality control
  - Need for standards
- Conflicting rights and duties
  - Tensions between duties to patients and to science
  - Anonymization and data linkage
- Need for normative international guidelines
  - Lack of normative guidelines
- Aims and goals of clinical proteomics
  - Aims of research
- Regulation
  - Informed Consent

Reviewer: Sebastian Porsdam Mann, Peter Vilmos Treit
Issues
- Tensions between duties to patients and to science
- Anonymization and data linkage
- Lack of international ethical framework/guidance
- Regulation
- Informed consent
- Need for standards
- Third party identifiability
- Individual relevance of results

ID 48757350: Boonen (2019)

Boonen Kurt, Hens Kristien and Menschaert Gerben ; Baggerman Geert ; Valkenborg Dirk ; Ertaylan Gökhan ;. 2019. "Beyond Genes: Re-Identifiability of Proteomic Data and Its Implications for Personalized Medicine.". Genes 10(9):682.
Abstract: The increasing availability of high throughput proteomics data provides us with opportunities as well as posing new ethical challenges regarding data privacy and re-identifiability of participants. Moreover, the fact that proteomics represents a level between the genotype and the phenotype further exacerbates the situation, introducing dilemmas related to publicly available data, anonymization, ownership of information and incidental findings. In this paper, we try to differentiate proteomics from genomics data and cover the ethical challenges related to proteomics data sharing. Finally, we give an overview of the proposed solutions and the outlook for future studies.

Narrative Summary: This article points out that consistent and ongoing increases in “depth (resolution) and size (population range) of proteomics studies not only allows significant scientific progress but also poses ethical challenges regarding personal privacy sharing…” The authors point out that much genomic information may now be deduced from proteomic studies. Where this is the case, the legal and ethical discussions, guidelines and regulations become directly relevant. The authors point to identifiability and personally sensitive data as defined by the GDPR and other regulatory instruments pertaining to genomic information but whose definitions likewise plausibly encompass some proteomic studies. The authors distinguish between the identifiability and therefore the privacy risk of different proteomic methodologies: “SRM has the lowest sensitive information content, followed by PRM, DDA and DIA.” More importantly, they point out that “[p]roteomics data is considered relatively safe for sharing traditionally, but today it consists of an ever larger amount of sequence information; this to an extent that is similar to genomic level data, where anonymization and privacy is recognized to be absolutely crucial… proteins (in the form of transcriptomics, proteomics and peptidomics) provide more biologically relevant information on the current state of the phenotype. Proteomes can also be acquired on the cellular up to the systemic level (organs, blood, urine andcerebrospinal fluid) and can therefore represent their origin, which makes proteomics indispensable in precision medicine. Proteomics is therefore not only a “watered-down” version of genomics privacy wise but in addition contains information on a person’s phenotype. Therefore, it should be treated with care and also presents new idiosyncratic challenges for its use in personalized medicine.”

The authors point out that not all proteomic data should be accorded the same status as genomics data, although some clearly should. Referring to Li et al. 2016, summarized above, the authors reiterated that “Li and colleagues used only the minor allelic frequencies of nsSNPs and adjusted the likelihood of identification by the global peptide false discovery

rate [36]. The analysis proved that enough nsSNPs from minor alleles can be detected in serum/plasma (up to 20) for identification, and this minimal requirement of SAPs will soon pose even less of a problem since the performance of LC–MS systems is increasing rapidly. The study also already pointed out some of the peculiarities of proteomics compared to genomics data. First of all, proteomics data is a subsample of the genome and the amount of identifiable data depends on the sample. Second, an additional layer of uncertainty is connected to peptide identifications, as every single peptide identification has a probability value attached to it and highly significant SAP identifications can be weighed more than less significant identifications. DDA, DIA, PRM and SRM all have theoretical frameworks to control the false discovery rate and have methods to assign confidence to a particular identification in an experiment. Third, the bioinformatic identification stage is the main cause of differences in interlaboratory reproducibility (hence the need for submission of data to public repositories)."

This need for submission of data to public repositories to check workflows and enhance reproducibility is a major concern for Boonen et al., since raw proteomic data uploaded to public databases has already been demonstrated to contain enough information to derive sufficient genetic loci for personal identification, even using only publicly available information and a surname.

Importantly, the authors note that "The discussion on the ethical aspects of proteomics can be picked up by the current discussion on genomics data and biobanking. We would like to emphasize that it will most likely not be possible to have one answer that would fit all situations (and study conditions) but an internationally recognized set of ethical guidelines to follow up would be a step forward. This would potentially assist the ethical committees advising personalized medicine and clinical proteomics studies."

Furthermore, the article points out that proteomics raises further unique ethical issues related to phenotypic information. "As we described above, it may be possible to obtain DNA information from proteins, which would immediately make all the ethical issues related to genetic research also relevant for proteomics. Moreover, the fact that also phenotypic information may be deduced from proteomics, makes this issue even more pertinent. Indeed, one of the reasons why privacy is important with regard to such information is the possibility that this information is misused by third parties such as employers and insurers. For the purposes that they would be interested in, phenotypic information is more interesting than genotypes alone."

The authors also touch upon, but do not discuss in depth, the issue of incidental findings. "A reason why complete anonymization of samples may be problematic (from an ethical point) is the fact that certain health information that is relevant to the research participants themselves may be discovered. How to deal with these incidental findings has been a hot topic in the debate regarding genomic data and the question will be even more pertinent in proteomics." Combining the two themes, the authors point out that "[a typical use of proteomics in such endeavors [integrated omics and wearable sensor data] is thelongitudinal follow up of urine or various types of blood samples. Such varied forms of data can provide insight in (a) health risks related to genetic, environmental and behavioral factors, (b) insights in molecular mechanisms associated with disease and (c) possible leads for new therapies. However,the data obtained also generates even more privacy issues concerning a person's health. This poses even harder ethical, practical and legal challenges. It will be possible to determine personal health risks (for instance based on an individual's genetic profile) while, at the same time, monitor changes in molecular pathways associated with these health risks. Although this opens up the possibility to go to a system of personalized prevention, it also creates an urgent need to establish an ethical framework for such studies that will try to tackle problems associated with the use of these technologies and the results

they will produce. In these cases, a framework needs to be established in which participants can be informed on actions to improve health or prevent disease. An important part of this ethical framework will pertain reporting (non-)incidental findings and risk factors. Increased knowledge of one's health can increase the mental burden of being responsible for one's own health, but risk factors and lifestyle recommendations should be balanced by the best knowledge on their effectiveness."

Finally, the authors touch upon the important question of data ownership and the economic value of aggregate proteomic data: "aggregated data would be a highly valuable economical commodity whose ownership would be less obvious and traded and used more easily. The challenges ahead are to determine the definition of sensitive and identifiable information within the proteomics datasets and whether this information can be removed or made inaccessible without significantly reducing the scientific quality."

The authors provide several specific recommendations: "Quantitative data extraction can easily be done on known non-SAPs, having a reliable feature detection algorithm being the only liability. Processed quantitative data can therefore be disseminated without privacy risks. Likewise, looking for proteins in online datasets and PTM analyses can be processed and filtered to leave out SAPs. This means that only analyses directly involving detecting and reporting SAPs in raw data should be scrutinized in assessing re-identifiability issues. Working towards consensus identification and quantification pipelines would address parts of this problem."

However, perhaps most importantly, "Personalized medicine, with its various data formats (genomics, proteomics, metabolomics, etc.), is a more challenging domain. Recently blockchain technology is being proposed [51,52] asa distributed electronic ledger for hosting health information. Blockchain technology allows us to create a distributed, transparent, independent and secure private information ledger where health data providers (individuals) are in control, own their information and can monitor access privileges as well as being informed about who accessed their information. Although, this technology is new in the health domain and currently only realized by a single start-up (Genomes.io) [53,54], it has potential for growth since it enables a data-driven marketplace to be created where users can receive tangible benefits for making their data accessible and immutable to the research organizations, application development community, pharmaceutical and consumer businesses. Blockchain alone does not solve the re-identifiability problem, however it addresses the issue of consent while simplifying and incentivizing data sharing in a secure and transparent manner. Currently the market leader is the KSI® blockchain technology stack developed by Guardtime, which is being used by NATO, the US Department of Defense, Lockheed Martin, Boeing, Ericsson, Telstra, SAP, GE and in Estonia where majority of the state data systems utilize the blockchain technology to enforce the integrity of government data and systems. The application of blockchain technology for personalized medicine (e.g., by the Estonian Genome Center, Tartu, Estonia) is currently being implemented."

The author's own conclusions deserve to be cited: "A consistent increase in both depth (resolution) and size (population range) of proteomics studies not only allows significant scientific progress but also poses ethical challenges regarding personal privacy sharing for where reproducibility is a fundamental pillar in science, the fact that it may be possible to deduce genomic information from proteomic data means discussions regarding privacy, sharing of results and regulations that are applicable to genomics may also become relevant for the proteomics field (not all researchers might be aware of this currently). Moreover, the fact that proteomics represents a level between the genotype and the phenotype may introduce even more intricate questions related to data access, ownership of information and incidental findings. We believe that trust in research is a precious

commodity to be protected. This means, on the one hand, that researchers should be made aware of these potential issues. Researchers should know that existing privacy regulations might also apply to proteomics research and be conscious about this when dealing with proteomics data. On the other hand, research participants have the right to know about these issues, both about what we know now and what we may know in the future. Consequently, consent procedures may need to be revised from time to time and platforms for dynamic consent may be set up
to allow for smooth communication between researcher and participants.
Therefore we envision the next generation of scientific enterprise is a highly collaborative environment where researchers recognize that they are entrusted with invaluable personal information, and research participants feel their data is safe, where demands of open science and the need for data protection are consolidated."

Reviewer: Sebastian Porsdam Mann, Peter Vilmos Treit
Themes

- Sensitive data/discrimination
  - Discrimination
  - Sensitivity of data/personal data
- Identifiability/privacy
  - Privacy
  - Identification/identifiability
  - Forensic use
- Incidental findings/reuse
  - Incidental findings/Secondary analysis
  - Uncertainty
  - Individual relevance of results
- Standards and quality control
  - Public databases: access and data integrity
  - Need for standards
  - Interoperability/inefficiencies
- Conflicting rights and duties
  - Tensions between duties to patients and to science
  - Anonymization and data linkage
  - Economic incentives for data sharing/undue inducement
  - Public v Private (profit)
  - Patient/data subject rights
  - Intellectual property
- Need for normative international guidelines
  - Lack of normative guidelines
  - Trust
  - International collaboration
- Aims and goals of clinical proteomics
  - Need for positive ethics beyond regulations
  - Need for discussion within field
- Benefits/Justice
  - Profit/benefit sharing
  - Data sharing
- Regulation
  - GDPR
  - Informed Consent
- Integration of new technologies and related fields
  - Evolution of technology
  - Complicating factors

Reviewer: Sebastian Porsdam Mann, Peter Vilmos Treit
Issues

- Discrimination
- Privacy
- Identification/identifiability
- Tensions between duties to patients and to science
- Profit/benefit sharing
- Data sharing
- Anonymization and data linkage
- Sensitivity of data/personal data
- Public databases: access and data integrity
- GDPR
- Incidental Findings/Secondary Analysis
- Uncertain data/evolution of technology
- Integration with other OMICs and emerging tech: complicating factors
- Lack of international ethical framework/guidance
- Trust
- Economic incentives for data sharing
- Private v Public (Profit, ownership)
- Regulation
- Informed consent
- Rights
- Intellectual Property
- Need for standards

ID 48757359: Critselis (2019)

Critselis Elena. 2019. "Impact of the General Data Protection Regulation on Clinical Proteomics Research". PROTEOMICS – Clinical Applications 13(2):1800199.
Abstract: Abstract The recently implemented General Data Protection Regulation (GDPR) has promising attributes for ensuring the protection of personal data collected and processed for clinical proteomic investigations. However, there exist ever increasing alarming concerns regarding its implications upon the future of clinical proteomics research both within and beyond the European Union. The main issues of concern regard GDPR legislative requirements for informed consent for study subjects? data collection and processing, data anonymization, and data storage and/or sharing, particularly in research areas which readily utilize databanks and biobanks, such as clinical proteomics investigations. The potential impacts of the aforementioned issues upon on-going and future clinical proteomics investigations are detailed, whilst recommendations for potentially resolving these emerging issues are proposed. Consensus between government, legislative, and research stakeholders, as well as impact assessments of final measures to be applied for medical research, is necessary so as to ensure the favorable perpetuation of clinical proteomics investigations and subsequent impact upon optimal patient health.

Narrative summary: Critselis 2019
This article introduces the EU 2018 GDPR and explains its direct relevance to clinical proteomics. It begins by explaining that “the overarching objectives of the legislative measures taken are: a) to provide rules for the protection and processing of personal data; b) to protect the fundamental rights and freedoms of individuals, particularly in relation to their personal data, and c) to ensure that personal data can move freely within the EU…” and that given the provisions inherent in the previous Data Protection Directive, the GDPR “explicitly plac[es] foremost priority on individuals’ related rights and needs.” The GDPR is most important in relation to the storage, processing, and sharing of data, as well as informed consent requirements. “Specific research areas anticipated to be most affected include those encompassing the use of research databanks[1] and biobanks,[3] such as

those often used within the context of clinical proteomics investigations." The author provides a visual summary of the most important GDPR-related issues for clinical proteomics research, reproduced below:

The author offers a number of specific legal solutions, including reliance on the 'necessary for research' exception, but underscores that it is not clear how far this exception will be accepted in court or politics. In summary, the author notes that "[s]pecifically, for clinical proteomics investigations to achieve their full potential, the current limitations posed in the collection, processing and use of data ought to be overcome. Currently, given the extraordinarily high penalties which may be incurred upon institutions and researchers for implementing clinical proteomics investigations which, albeit even unintentionally, do not and/or cannot comply with current GDPR requirements, research organizations and study investigators alike must rely upon the careful assessment and further guidance from their respective Institutional Ethical Review Boards for ensuring the compliance of individual investigations with the complex requirements set forth by the GDPR. However, such a practice does not automatically render researchers immune to either the legal responsibilities and/or related fines which may be posed should IRB falter in providing sound guidance on any of the complex and ever evolving issues set forth by the GDPR. Nonetheless, there exists an urgent need for government, legislative, and research stakeholders to reach a consensus of the issues posed, including a careful impact assessment of final measures to be applied for medical research,[2] so as to secure optimal clinical proteomics (and in fact, any type of clinical molecular profiling) research outcomes which may ultimately optimize the health of European (and beyond) citizens.

Reviewer: Sebastian Porsdam Mann, Peter Vilmos Treit
Themes

- Sensitive data/discrimination
  - Discrimination
  - Sensitivity of data/personal data
- Identifiability/privacy
  - Privacy
  - Identification/identifiability
- Incidental findings/reuse
  - Incidental findings/Secondary analysis
  - Uncertainty
- Standards and quality control
  - Public databases: access and data integrity
  - Need for standards
- Conflicting rights and duties
  - Tensions between duties to patients and to science
  - Anonymization and data linkage
  - Public v Private (profit)
  - Patient/data subject rights
  - Intellectual property
- Need for normative international guidelines
  - Lack of normative guidelines
  - International collaboration
- Aims and goals of clinical proteomics
  - Need for positive ethics beyond regulations
  - Need for discussion within field
- Benefits/Justice
  - Profit/benefit sharing
  - Data sharing
- Regulation
  - GDPR

o Informed Consent
• Integration of new technologies and related fields
o Evolution of technology
o Complicating factors

Reviewer: Sebastian Porsdam Mann, Peter Vilmos Treit
Issues
• Discrimination
• Privacy
• Identification/identifiability
• Tensions between duties to patients and to science
• Data sharing
• Anonymization and data linkage
• Sensitivity of data/personal data
• GDPR
• Incidental Findings/Secondary Analysis
• Lack of international ethical framework/guidance
• Private v Public (Profit, ownership)
• Regulation
• Informed consent
• Rights

ID 48757349: Gupta (2014)

Gupta Shabarni, Venkatesh Apoorva and Ray Sandipan ; Srivastava Sanjeeva ;. 2014. "Challenges and prospects for biomarker research: a current perspective from the developing world.". Biochimica et Biophysica Acta 1844(5):899-908.
Abstract: Abstract Majority of deaths due to communicable and non-communicable diseases occur in the low and middle-income nations (LMNs), mainly due to the lack of early diagnoses and timely treatments. In such a scenario, biomarkers serve as an indispensible resource that can be used as indicators of biological processes, specific disease conditions or response to therapeutic interventions. Evaluation, diagnosis and management of diseases in developing world by following/extrapolating the findings obtained on the basis of the research work involving only the populations from the developed countries, could often be highly misleading due to existence of diverse patterns of diseases in developing countries compared to the developed world. Biomarker candidates identified from high-throughput integrated omics technologies have promising potential; however, their actual clinical applications are found to be limited, primarily due to the challenges of disease heterogeneity and pre-analytical variability associated with the biomarker discovery pipeline. Additionally, in the developing world, economic crunches, lack of awareness and education, paucity of biorepositories, enormous diversities in socio-epidemiological background, ethnicity, lifestyle, diet, exposure to various environmental risk factors and infectious agents, and ethical and social issues also cumulatively hinder biomarker discovery ventures. Establishment of standard operating procedures, comprehensive data repositories and exchange of scientific findings are crucial for reducing the variability and fragmentation of data. This review highlights the challenges associated with the discovery, validation and translational phases of biomarker research in LMNs with some of their amenable solutions and future prospects. This article is part of a Special Issue entitled: Biomarkers: A Proteomic Challenge.

Narrative summary: This article pointed to several overarching normative issues in clinical proteomics with a special focus on international collaboration and the needs and specific contexts of the Global South. The authors point to the 10/90 gap and several issues of

affordability, lack of interoperability, disparities of human and technological capital, access issues to knowledge, data and journal articles, as well as educational opportunities. The study points out that greater international collaboration could enhance proteomics research, since a vast heterogenous part of the global human variability in proteomes, and their correlations with more extreme and wide-ranging environmental and socioeconomic factors, could be a great boon for proteomics generally. However, the authors call for more help from the Global North, especially in technology and educational transfer as well as in the arena of intellectual property rights.

Reviewer: Sebastian Porsdam Mann, Peter Vilmos Treit
Themes

- Sensitive data/discrimination
  - Discrimination
  - Sensitivity of data/personal data
- Identifiability/privacy
  - Privacy
  - Identification/identifiability
- Incidental findings/reuse
  - Incidental findings/Secondary analysis
  - Uncertainty
  - Individual relevance of results
- Standards and quality control
  - Public databases: access and data integrity
  - Need for standards
  - Interoperability/inefficiencies
- Conflicting rights and duties
  - Public v Private (profit)
  - Patient/data subject rights
  - Intellectual property
- Need for normative international guidelines
  - Lack of normative guidelines
  - Trust
  - International collaboration
  - Public awareness/public opinion
  - Social consequences
- Aims and goals of clinical proteomics
  - Funding priorities
  - Aims of research
  - Need for positive ethics beyond regulations
  - Need for discussion within field
- Benefits/Justice
  - Profit/benefit sharing
  - Data sharing
  - Access for low-and-middle-income countries/researchers
  - Databases not globally representative
  - Solidarity
  - Altruism
  - Fairness
  - Authority/power
- Regulation
  - Informed Consent
- Integration of new technologies and related fields
  - Evolution of technology
  - Complicating factors

Reviewer: Sebastian Porsdam Mann, Peter Vilmos Treit
Issues
- Discrimination
- Privacy
- Identification/identifiability
- Profit/benefit sharing
- Data sharing
- Sensitivity of data/personal data
- Public databases: access and data integrity
- Incidental Findings/Secondary Analysis
- Uncertain data/evolution of technology
- Integration with other OMICs and emerging tech: complicating factors
- Lack of international ethical framework/guidance
- Private v Public (Profit, ownership)
- Regulation
- Informed consent
- Rights
- Intellectual Property
- Need for standards
- Access for low-and-middle middle-income countries/researchers
- Funding priorities/lack of funding
- International collaboration
- Databases and research topics not representative globally

ID 48757347: Holmes (2016)

Holmes Christina, Carlson Siobhan M and McDonald Fiona ; Jones Mavis ; Graham Janice E;. 2016. "Exploring the post-genomic world: differing explanatory and manipulatory functions of post-genomic sciences". New Genetics and Society 35(1):49-68.
Abstract: Richard Lewontin proposed that the ability of a scientific field to create a narrative for public understanding garners it social relevance. This article applies Lewontin's conceptual framework of the functions of science (manipulatory and explanatory) to compare and explain the current differences in perceived societal relevance of genetics/genomics and proteomics. We provide three examples to illustrate the social relevance and strong cultural narrative of genetics/genomics for which no counterpart exists for proteomics. We argue that the major difference between genetics/genomics and proteomics is that genomics has a strong explanatory function, due to the strong cultural narrative of heredity. Based on qualitative interviews and observations of proteomics conferences, we suggest that the nature of proteins, lack of public understanding, and theoretical complexity exacerbates this difference for proteomics. Lewontin's framework suggests that social scientists may find that omics sciences affect social relations in different ways than past analyses of genetics.

Narrative summary: This article applies Lewontin’s conceptual framework of the functions of science (manipulatory and explanatory) to compare and explain the current (2015) differences in societal relevance of genomics and proteomics, based on 35 interviews from proteomics scientists. The authors argue that genomics has a greater explanatory function due to the public’s understanding of heredity through DTC goods, and equality in terms of false racial and genetic differences. The authors argue that proteomcs is more difficult to understand due to the theoretical complexity of proteins c.f. DNA (4 bases vs. 20 AAs, relatively static genotype vs. Dynamic phenotype and more complicated tools to study proteins). This weaker explanatory function is not static and may develop. The authors conclude that the potential manipulatory functions of proteomics are greater but yet to be

realised, seeing the genotype as a blueprint nevertheless still needs to be expressed, and that through nutrigenomics, proteomics can build on decades of existing biochemical knowledge of metabolism to survey proteins on a massive scale. This would aid the explanatory functions of proteomics, bringing it to the public.

Reviewer: Sebastian Porsdam Mann, Peter Vilmos Treit
Themes
• Need for normative international guidelines
o Trust
o Public awareness/public opinion

Reviewer: Sebastian Porsdam Mann, Peter Vilmos Treit
Issues
• Trust
• Public awareness/public opinion

ID 48757345: Jackson (2010)

Jackson David and Banks Rosamonde E. 2010. "Banking of clinical samples for proteomic biomarker studies: a consideration of logistical issues with a focus on pre-analytical variation.". Proteomics Clinical Applications 4(3):250-270.
Abstract: Biobanks with their collections of clinical samples and data are essential resources for the success of clinical proteomics in delivering and validating candidate biomarkers. Samples must be banked in a manner that allows maximum subsequent compatibility with analytical techniques and additionally many critical factors must be taken into account when establishing a biobank or selecting samples from a biobank. These include logistical, ethical, legal and security issues and, very importantly, steps to minimise any pre-analytical variability introduced N.B. This is the ‘report' output of the EPPI-Reviewer Beta Web platform which we used to conduct thematic analysis. It consists of a title, abstract, narrative summary (ranging from short to long) and a tree of themes with issues grouped underneath them. We generated these (except for the abstract) following the methodological recommendations cited in the methods section. This is all supplementary material and part of data extraction.

ID 48757562: Beck (2004)

Beck Norbert. 2004. "Proteomics in pathology, research and practice: ethical considerations". Pathology - Research and Practice 200(2):179-180.
Abstract: A researcher should always seek advice from his responsible ethics committee. Usually, advisory discussions with physicians doing research are not problematic in ethical terms when they want to examine biological material obtained, especially, when the material is no longer needed, the material will be used anonymized, no individualizing genes will be examined, the aims of research are not disputed in ethical terms, research is not expected to yield results of individual relevance to the person affected, there is no indication that the person affected will object to research and the expenditure for obtaining the individual consent is excessively high.

Narrative summary: Points out a number of general issues related to human samples. Only distinction made is that proteomics may involve smaller quantities of material than other study methodologies and therefore may involve fewer ethical issues.

Reviewer: Sebastian Porsdam Mann, Peter Vilmos Treit
Themes
- Identifiability/privacy
  - Third party identifiability
- Incidental findings/reuse
  - Individual relevance of results
- Standards and quality control
  - Need for standards
- Conflicting rights and duties
  - Tensions between duties to patients and to science
  - Anonymization and data linkage
- Need for normative international guidelines
  - Lack of normative guidelines
- Aims and goals of clinical proteomics
  - Aims of research
- Regulation
  - Informed Consent

Reviewer: Sebastian Porsdam Mann, Peter Vilmos Treit
Issues
- Tensions between duties to patients and to science
- Anonymization and data linkage
- Lack of international ethical framework/guidance
- Regulation
- Informed consent
- Need for standards
- Third party identifiability
- Individual relevance of results

ID 48757350: Boonen (2019)

Boonen Kurt, Hens Kristien and Menschaert Gerben ; Baggerman Geert ; Valkenborg Dirk ; Ertaylan Gökhan ;. 2019. "Beyond Genes: Re-Identifiability of Proteomic Data and Its Implications for Personalized Medicine.". Genes 10(9):682.
Abstract: The increasing availability of high throughput proteomics data provides us with opportunities as well as posing new ethical challenges regarding data privacy and re-identifiability of participants. Moreover, the fact that proteomics represents a level between the genotype and the phenotype further exacerbates the situation, introducing dilemmas related to publicly available data, anonymization, ownership of information and incidental findings. In this paper, we try to differentiate proteomics from genomics data and cover the ethical challenges related to proteomics data sharing. Finally, we give an overview of the proposed solutions and the outlook for future studies.

Narrative Summary: This article points out that consistent and ongoing increases in “depth (resolution) and size (population range) of proteomics studies not only allows significant scientific progress but also poses ethical challenges regarding personal privacy sharing…” The authors point out that much genomic information may now be deduced from proteomic studies. Where this is the case, the legal and ethical discussions, guidelines and regulations become directly relevant. The authors point to identifiability and personally sensitive data as defined by the GDPR and other regulatory instruments pertaining to genomic information but whose definitions likewise plausibly encompass some proteomic studies. The authors distinguish between the identifiability and therefore the privacy risk of different proteomic methodologies: “SRM has the lowest sensitive information content, followed by

PRM, DDA and DIA." More importantly, they point out that "[p]roteomics data is considered relatively safe for sharing traditionally, but today it consists of an ever larger amount of sequence information; this to an extent that is similar to genomic level data, where anonymization and privacy is recognized to be absolutely crucial… proteins (in the form of transcriptomics, proteomics and peptidomics) provide more biologically relevant information on the current state of the phenotype. Proteomes can also be acquired on the cellular up to the systemic level (organs, blood, urine and cerebrospinal fluid) and can therefore represent their origin, which makes proteomics indispensable in precision medicine. Proteomics is therefore not only a "watered-down" version of genomics privacy wise but in addition contains information on a person's phenotype. Therefore, it should be treated with care and also presents new idiosyncratic challenges for its use in personalized medicine."

The authors point out that not all proteomic data should be accorded the same status as genomics data, although some clearly should. Referring to Li et al. 2016, summarized above, the authors reiterated that "Li and colleagues used only the minor allelic frequencies of nsSNPs and adjusted the likelihood of identification by the global peptide false discovery rate [36]. The analysis proved that enough nsSNPsfrom minor alleles can be detected in serum/plasma (up to 20) for identification, and this minimal requirement of SAPs will soon pose even less of a problem since the performance of LC–MS systems is increasing rapidly. The study also already pointed out some of the peculiarities of proteomics compared to genomics data. First of all, proteomics data is a subsample of the genome and the amount of identifiable data depends on the sample. Second, an additional layer of uncertainty is connected to peptide identifications, as every single peptide identification has a probability value attached to it and highly significant SAP identifications can be weighed more than less significant identifications.DDA, DIA, PRM and SRM all have theoretical frameworks to control the false discovery rate and have methods to assign confidence to a particular identification in an experiment. Third, the bioinformatic identification stage is the main cause of differences in interlaboratory reproducibility (hence the need for submission of data to public repositories)."

This need for submission of data to public repositories to check workflows and enhance reproducibility is a major concern for Boonen et al., since raw proteomic data uploaded to public databases has already been demonstrated to contain enough information to derive sufficient genetic loci for personal identification, even using only publicly available information and a surname.

Importantly, the authors note that "The discussion on the ethical aspects of proteomics can be picked up by the current discussion on genomics data and biobanking. We would like to emphasize that it will most likely not be possible to have one answer that would fit all situations (and study conditions) but an internationally recognized set of ethical guidelines to follow up would be a step forward. This would potentially assist the ethical committees advising personalized medicine and clinical proteomics studies."

Furthermore, the article points out that proteomics raises further unique ethical issues related to phenotypic information. "As we described above, it may be possible to obtain DNA information from proteins, which would immediately make all the ethical issues related to genetic research also relevant for proteomics. Moreover, the fact that also phenotypic information may be deduced from proteomics, makes this issue even more pertinent. Indeed, one of the reasons why privacy is important with regard to such information is the possibility that this information is misused by third parties such as employers and insurers. For the purposes that they would be interested in, phenotypic information is more interesting than genotypes alone."

The authors also touch upon, but do not discuss in depth, the issue of incidental findings. “A reason why complete anonymization of samples may be problematic (from an ethical point) is
the fact that certain health information that is relevant to the research participants themselves may be
discovered. How to deal with these incidental findings has been a hot topic in the debate regarding genomic data and the question will be even more pertinent in proteomics.” Combining the two themes, the authors point out that “[a typical use of proteomics in such endeavors [integrated omics and wearable sensor data] is thelongitudinal follow up of urine or various types of blood samples. Such varied forms of data can provide insight in (a) health risks related to genetic, environmental and behavioral factors, (b) insights in molecular mechanisms associated with disease and (c) possible leads for new therapies. However, the data obtained also generates even more privacy issues concerning a person’s health. This poses
even harder ethical, practical and legal challenges. It will be possible to determine personal health risks (for instance based on an individual’s genetic profile) while, at the same time, monitor changes in molecular pathways associated with these health risks. Although this opens up the possibility to go to a system of personalized prevention, it also creates an urgent need to establish an ethical framework for such studies that will try to tackle problems associated with the use of these technologies and the results they will produce. In these cases, a framework needs to be established in which participants can be informed on actions to improve health or prevent disease. An important part of this ethical framework will pertain reporting (non-)incidental findings and risk factors. Increased knowledge of one’s health can increase the mental burden of being responsible for one’s own health, but risk factors and lifestyle recommendations should be balanced by the best knowledge on their effectiveness.”

Finally, the authors touch upon the important question of data ownership and the economic value of aggregate proteomic data: “aggregated data would be a highly valuable economical commodity whose ownership would be less obvious and traded and used more easily. The challenges ahead are to determine the definition of sensitive and identifiable information within the proteomics datasets and whether this information can be removed or made inaccessible without significantly reducing the scientific quality.”

The authors provide several specific recommendations: “Quantitative data extraction can easily be done on known non-SAPs, having a reliable feature detection algorithm being the only liability. Processed quantitative data can therefore be disseminated without privacy risks.
Likewise, looking for proteins in online datasets and PTM analyses can be processed and filtered to leave out SAPs. This means that only analyses directly involving detecting and reporting SAPs in raw data should be scrutinized in assessing re-identifiability issues. Working towards consensus identification and quantification pipelines would address parts of this problem.”

However, perhaps most importantly, “Personalized medicine, with its various data formats (genomics, proteomics, metabolomics, etc.), is a more challenging domain. Recently blockchain technology is being proposed [51,52] asa distributed electronic ledger for hosting health information. Blockchain technology allows us to create a distributed, transparent, independent and secure private information ledger where health data providers (individuals) are in control, own their information and can monitor access privileges as
well as being informed about who accessed their information. Although, this technology is new in the health domain and currently only realized by a single start-up (Genomes.io) [53,54], it has potential for growth since it enables a data-driven marketplace to be created where users can receive tangible benefits for making their data accessible and immutable to

the research organizations, application development community, pharmaceutical and consumer businesses. Blockchain alone does not solve the re-identifiability problem, however it addresses the issue of consent while simplifying and incentivizing data sharing in a secure and transparent manner. Currently the market leader is the KSI® blockchain technology stack developed by Guardtime, which is being used by NATO, the US Department of Defense, Lockheed Martin, Boeing, Ericsson, Telstra, SAP, GE and in Estonia where majority of the state data systems utilize the blockchain technology to enforce the integrity of government data and systems. The application of blockchain technology for personalized medicine (e.g., by the Estonian Genome Center, Tartu, Estonia) is currently being implemented."

The author's own conclusions deserve to be cited: "A consistent increase in both depth (resolution) and size (population range) of proteomics studies not only allows significant scientific progress but also poses ethical challenges regarding personal
privacy sharing for where reproducibility is a fundamental pillar in science, the fact that it may be
possible to deduce genomic information from proteomic data means discussions regarding privacy, sharing of results and regulations that are applicable to genomics may also become relevant for the proteomics field (not all researchers might be aware of this currently). Moreover, the fact that proteomics represents a level between the genotype and the phenotype may introduce even more intricate questions related to data access, ownership of information and incidental findings. We believe that trust in research is a precious commodity to be protected. This means, on the one hand, that researchers should be made aware of these potential issues. Researchers should know that existing privacy regulations might also apply to proteomics research and be conscious about this when dealing with proteomics data. On the other hand, research participants have the right to know about these issues, both about what we know now and what we may know in the future. Consequently, consent procedures may need to be revised from time to time and platforms for dynamic consent may be set up
to allow for smooth communication between researcher and participants.
Therefore we envision the next generation of scientific enterprise is a highly collaborative environment where researchers recognize that they are entrusted with invaluable personal information, and research participants feel their data is safe, where demands of open science and the need for data protection are consolidated."

Reviewer: Sebastian Porsdam Mann, Peter Vilmos Treit
Themes
- Sensitive data/discrimination
  - Discrimination
  - Sensitivity of data/personal data
- Identifiability/privacy
  - Privacy
  - Identification/identifiability
  - Forensic use
- Incidental findings/reuse
  - Incidental findings/Secondary analysis
  - Uncertainty
  - Individual relevance of results
- Standards and quality control
  - Public databases: access and data integrity
  - Need for standards
  - Interoperability/inefficiencies
- Conflicting rights and duties
  - Tensions between duties to patients and to science

- o Anonymization and data linkage
- o Economic incentives for data sharing/undue inducement
- o Public v Private (profit)
- o Patient/data subject rights
- o Intellectual property
- • Need for normative international guidelines
- o Lack of normative guidelines
- o Trust
- o International collaboration
- • Aims and goals of clinical proteomics
- o Need for positive ethics beyond regulations
- o Need for discussion within field
- • Benefits/Justice
- o Profit/benefit sharing
- o Data sharing
- • Regulation
- o GDPR
- o Informed Consent
- • Integration of new technologies and related fields
- o Evolution of technology
- o Complicating factors

Reviewer: Sebastian Porsdam Mann, Peter Vilmos Treit
Issues

- Discrimination
- Privacy
- Identification/identifiability
- Tensions between duties to patients and to science
- Profit/benefit sharing
- Data sharing
- Anonymization and data linkage
- Sensitivity of data/personal data
- Public databases: access and data integrity
- GDPR
- Incidental Findings/Secondary Analysis
- Uncertain data/evolution of technology
- Integration with other OMICs and emerging tech: complicating factors
- Lack of international ethical framework/guidance
- Trust
- Economic incentives for data sharing
- Private v Public (Profit, ownership)
- Regulation
- Informed consent
- Rights
- Intellectual Property
- Need for standards

ID 48757359: Critselis (2019)

Critselis Elena. 2019. "Impact of the General Data Protection Regulation on Clinical Proteomics Research". PROTEOMICS – Clinical Applications 13(2):1800199.
Abstract: Abstract The recently implemented General Data Protection Regulation (GDPR) has promising attributes for ensuring the protection of personal data collected and processed for clinical proteomic investigations. However, there exist ever increasing

alarming concerns regarding its implications upon the future of clinical proteomics research both within and beyond the European Union. The main issues of concern regard GDPR legislative requirements for informed consent for study subjects? data collection and processing, data anonymization, and data storage and/or sharing, particularly in research areas which readily utilize databanks and biobanks, such as clinical proteomics investigations. The potential impacts of the aforementioned issues upon on-going and future clinical proteomics investigations are detailed, whilst recommendations for potentially resolving these emerging issues are proposed. Consensus between government, legislative, and research stakeholders, as well as impact assessments of final measures to be applied for medical research, is necessary so as to ensure the favorable perpetuation of clinical proteomics investigations and subsequent impact upon optimal patient health.

Narrative summary: Critselis 2019
This article introduces the EU 2018 GDPR and explains its direct relevance to clinical proteomics. It begins by explaining that "the overarching objectives of the legislative measures taken are: a) to provide rules for the protection and processing of personal data; b) to protect the fundamental rights and freedoms of individuals, particularly in relation to their personal data, and c) to ensure that personal data can move freely within the EU…" and that given the provisions inherent in the previous Data Protection Directive, the GDPR "explicitly plac[es] foremost priority on individuals' related rights and needs." The GDPR is most important in relation to the storage, processing, and sharing of data, as well as informed consent requirements. "Specific research areas anticipated to be most affected include those encompassing the use of research databanks[1] and biobanks,[3] such as those often used within the context of clinical proteomics investigations." The author provides a visual summary of the most important GDPR-related issues for clinical proteomics research, reproduced below:

The author offers a number of specific legal solutions, including reliance on the 'necessary for research' exception, but underscores that it is not clear how far this exception will be accepted in court or politics. In summary, the author notes that "[s]pecifically, for clinical proteomics investigations to achieve their full potential, the current limitations posed in the collection, processing and use of data ought to be overcome. Currently, given the extraordinarily high penalties which may be incurred upon institutions and researchers for implementing clinical proteomics investigations which, albeit even unintentionally, do not and/or cannot comply with current GDPR requirements, research organizations and study investigators alike must rely upon the careful assessment and further guidance from their respective Institutional Ethical Review Boards for ensuring the compliance of individual investigations with the complex requirements set forth by the GDPR. However, such a practice does not automatically render researchers immune to either the legal responsibilities and/or related fines which may be posed should IRB falter in providing sound guidance on any of the complex and ever evolving issues set forth by the GDPR. Nonetheless, there exists an urgent need for government, legislative, and research stakeholders to reach a consensus of the issues posed, including a careful impact assessment of final measures to be applied for medical research,[2] so as to secure optimal clinical proteomics (and in fact, any type of clinical molecular profiling) research outcomes which may ultimately optimize the health of European (and beyond) citizens.

Reviewer: Sebastian Porsdam Mann, Peter Vilmos Treit
Themes
- Sensitive data/discrimination
  - Discrimination
  - Sensitivity of data/personal data
- Identifiability/privacy
  - Privacy
  - Identification/identifiability

• Incidental findings/reuse
o Incidental findings/Secondary analysis
o Uncertainty
• Standards and quality control
o Public databases: access and data integrity
o Need for standards
• Conflicting rights and duties
o Tensions between duties to patients and to science
o Anonymization and data linkage
o Public v Private (profit)
o Patient/data subject rights
o Intellectual property
• Need for normative international guidelines
o Lack of normative guidelines
o International collaboration
• Aims and goals of clinical proteomics
o Need for positive ethics beyond regulations
o Need for discussion within field
• Benefits/Justice
o Profit/benefit sharing
o Data sharing
• Regulation
o GDPR
o Informed Consent
• Integration of new technologies and related fields
o Evolution of technology
o Complicating factors

Reviewer: Sebastian Porsdam Mann, Peter Vilmos Treit
Issues
• Discrimination
• Privacy
• Identification/identifiability
• Tensions between duties to patients and to science
• Data sharing
• Anonymization and data linkage
• Sensitivity of data/personal data
• GDPR
• Incidental Findings/Secondary Analysis
• Lack of international ethical framework/guidance
• Private v Public (Profit, ownership)
• Regulation
• Informed consent
• Rights

ID 48757349: Gupta (2014)

Gupta Shabarni, Venkatesh Apoorva and Ray Sandipan ; Srivastava Sanjeeva ;. 2014. "Challenges and prospects for biomarker research: a current perspective from the developing world.". Biochimica et Biophysica Acta 1844(5):899-908.
Abstract: Abstract Majority of deaths due to communicable and non-communicable diseases occur in the low and middle-income nations (LMNs), mainly due to the lack of early diagnoses and timely treatments. In such a scenario, biomarkers serve as an indispensible resource that can be used as indicators of biological processes, specific disease conditions

or response to therapeutic interventions. Evaluation, diagnosis and management of diseases in developing world by following/extrapolating the findings obtained on the basis of the research work involving only the populations from the developed countries, could often be highly misleading due to existence of diverse patterns of diseases in developing countries compared to the developed world. Biomarker candidates identified from high-throughput integrated omics technologies have promising potential; however, their actual clinical applications are found to be limited, primarily due to the challenges of disease heterogeneity and pre-analytical variability associated with the biomarker discovery pipeline. Additionally, in the developing world, economic crunches, lack of awareness and education, paucity of biorepositories, enormous diversities in socio-epidemiological background, ethnicity, lifestyle, diet, exposure to various environmental risk factors and infectious agents, and ethical and social issues also cumulatively hinder biomarker discovery ventures. Establishment of standard operating procedures, comprehensive data repositories and exchange of scientific findings are crucial for reducing the variability and fragmentation of data. This review highlights the challenges associated with the discovery, validation and translational phases of biomarker research in LMNs with some of their amenable solutions and future prospects. This article is part of a Special Issue entitled: Biomarkers: A Proteomic Challenge.

Narrative summary: This article pointed to several overarching normative issues in clinical proteomics with a special focus on international collaboration and the needs and specific contexts of the Global South. The authors point to the 10/90 gap and several issues of affordability, lack of interoperability, disparities of human and technological capital, access issues to knowledge, data and journal articles, as well as educational opportunities. The study points out that greater international collaboration could enhance proteomics research, since a vast heterogenous part of the global human variability in proteomes, and their correlations with more extreme and wide-ranging environmental and socioeconomic factors, could be a great boon for proteomics generally. However, the authors call for more help from the Global North, especially in technology and educational transfer as well as in the arena of intellectual property rights.

Reviewer: Sebastian Porsdam Mann, Peter Vilmos Treit
Themes

- Sensitive data/discrimination
  - Discrimination
  - Sensitivity of data/personal data
- Identifiability/privacy
  - Privacy
  - Identification/identifiability
- Incidental findings/reuse
  - Incidental findings/Secondary analysis
  - Uncertainty
  - Individual relevance of results
- Standards and quality control
  - Public databases: access and data integrity
  - Need for standards
  - Interoperability/inefficiencies
- Conflicting rights and duties
  - Public v Private (profit)
  - Patient/data subject rights
  - Intellectual property
- Need for normative international guidelines
  - Lack of normative guidelines
  - Trust

- o International collaboration
- o Public awareness/public opinion
- o Social consequences
- • Aims and goals of clinical proteomics
- o Funding priorities
- o Aims of research
- o Need for positive ethics beyond regulations
- o Need for discussion within field
- • Benefits/Justice
- o Profit/benefit sharing
- o Data sharing
- o Access for low-and-middle-income countries/researchers
- o Databases not globally representative
- o Solidarity
- o Altruism
- o Fairness
- o Authority/power
- • Regulation
- o Informed Consent
- • Integration of new technologies and related fields
- o Evolution of technology
- o Complicating factors

Reviewer: Sebastian Porsdam Mann, Peter Vilmos Treit
Issues

- • Discrimination
- • Privacy
- • Identification/identifiability
- • Profit/benefit sharing
- • Data sharing
- • Sensitivity of data/personal data
- • Public databases: access and data integrity
- • Incidental Findings/Secondary Analysis
- • Uncertain data/evolution of technology
- • Integration with other OMICs and emerging tech: complicating factors
- • Lack of international ethical framework/guidance
- • Private v Public (Profit, ownership)
- • Regulation
- • Informed consent
- • Rights
- • Intellectual Property
- • Need for standards
- • Access for low-and-middle middle-income countries/researchers
- • Funding priorities/lack of funding
- • International collaboration
- • Databases and research topics not representative globally

ID 48757347: Holmes (2016)

Holmes Christina, Carlson Siobhan M and McDonald Fiona ; Jones Mavis ; Graham Janice E;. 2016. "Exploring the post-genomic world: differing explanatory and manipulatory functions of post-genomic sciences". New Genetics and Society 35(1):49-68.
Abstract: Richard Lewontin proposed that the ability of a scientific field to create a narrative for public understanding garners it social relevance. This article applies Lewontin's

conceptual framework of the functions of science (manipulatory and explanatory) to compare and explain the current differences in perceived societal relevance of genetics/genomics and proteomics. We provide three examples to illustrate the social relevance and strong cultural narrative of genetics/genomics for which no counterpart exists for proteomics. We argue that the major difference between genetics/genomics and proteomics is that genomics has a strong explanatory function, due to the strong cultural narrative of heredity. Based on qualitative interviews and observations of proteomics conferences, we suggest that the nature of proteins, lack of public understanding, and theoretical complexity exacerbates this difference for proteomics. Lewontin's framework suggests that social scientists may find that omics sciences affect social relations in different ways than past analyses of genetics.

Narrative summary: This article applies Lewontin’s conceptual framework of the functions of science (manipulatory and explanatory) to compare and explain the current (2015) differences in societal relevance of genomics and proteomics, based on 35 interviews from proteomics scientists. The authors argue that genomics has a greater explanatory function due to the public’s understanding of heredity through DTC goods, and equality in terms of false racial and genetic differences. The authors argue that proteomcs is more difficult to understand due to the theoretical complexity of proteins c.f. DNA (4 bases vs. 20 AAs, relatively static genotype vs. Dynamic phenotype and more complicated tools to study proteins). This weaker explanatory function is not static and may develop. The authors conclude that the potential manipulatory functions of proteomics are greater but yet to be realised, seeing the genotype as a blueprint nevertheless still needs to be expressed, and that through nutrigenomics, proteomics can build on decades of existing biochemical knowledge of metabolism to survey proteins on a massive scale. This would aid the explanatory functions of proteomics, bringing it to the public.

Reviewer: Sebastian Porsdam Mann, Peter Vilmos Treit
Themes
- Need for normative international guidelines
  - Trust
  - Public awareness/public opinion

Reviewer: Sebastian Porsdam Mann, Peter Vilmos Treit
Issues
- Trust
- Public awareness/public opinion

ID 48757345: Jackson (2010)

Jackson David and Banks Rosamonde E. 2010. "Banking of clinical samples for proteomic biomarker studies: a consideration of logistical issues with a focus on pre-analytical variation.". Proteomics Clinical Applications 4(3):250-270.
Abstract: Biobanks with their collections of clinical samples and data are essential resources for the success of clinical proteomics in delivering and validating candidate biomarkers. Samples must be banked in a manner that allows maximum subsequent compatibility with analytical techniques and additionally many critical factors must be taken into account when establishing a biobank or selecting samples from a biobank. These include logistical, ethical, legal and security issues and, very importantly, steps to minimise any pre-analytical variability introduced through sample processing and handling (technical effects). The inherent variation present within the samples must also be taken into account. In this review, we examine the impact of these factors and issues to be faced when banking samples with a particular focus on sources of pre-analytical variation, which must be rigorously controlled

and recorded. It is encouraging that several initiatives are now addressing such key issues and these are also discussed.

Narrative summary: In this proteomics-specific review, the effects of pre-analytical steps and general logistics of storing samples in biobanks are explored. From an ethical and legal perspective, the paper highlights the difficulty in that samples might be used for a different purpose as originally intended, as well as different national and multinational codes of conduct that protect both the donor and researcher wishing to analyze the sample. Furthermore, the paper highlights potential steps to ensure secure sample and data collection (clinical metadata, demographic and storage data), storage and access. Measures taken here should allow for avoiding biases, adherence to good clinical practice and compatibility with clinical trials. The paper's main focus is on pre-analytical variation, and the plethora of ways methods relating to samples need to be standardised in order to adhere to clinical standards. These areas include steps technical steps such sample collection, storage, processing and processing, parameters that vary widely for the studied material (biofluid / tissue). The paper also highlights the biological variation as something to keep in mind, and that this is an active area of research where standardisation of sample protocols and solid systematic reviews are thus far lacking.

Reviewer: Sebastian Porsdam Mann, Peter Vilmos Treit
Themes
- Standards and quality control
  - Public databases: access and data integrity
  - Need for standards
  - Interoperability/inefficiencies
- Need for normative international guidelines
  - Lack of normative guidelines
  - International collaboration
- Regulation

Reviewer: Sebastian Porsdam Mann, Peter Vilmos Treit
Issues
- Public databases: access and data integrity
- Lack of international ethical framework/guidance
- Regulation
- Need for standards

ID 48757358: Laatsch (2014)

Laatsch Chelsea N, Durbin-Johnson Blythe P; Rocke David M; Mukwana Sophie and Newland Abby B; Flagler Michael J; Davis Michael G; Eigenheer Richard A; Phinney Brett S; Rice Robert H;. 2014. "Human hair shaft proteomic profiling: individual differences, site specificity and cuticle analysis". PeerJ 2:e506-e506.
Abstract: Hair from different individuals can be distinguished by physical properties. Although some data exist on other species, examination of the individual molecular differences within the human hair shaft has not been thoroughly investigated. Shotgun proteomic analysis revealed considerable variation in profile among samples from Caucasian, African-American, Kenyan and Korean subjects. Within these ethnic groups, prominent keratin proteins served to distinguish individual profiles. Differences between ethnic groups, less marked, relied to a large extent on levels of keratin associated proteins. In samples from Caucasian subjects, hair shafts from axillary, beard, pubic and scalp

regions exhibited distinguishable profiles, with the last being most different from the others. Finally, the profile of isolated hair cuticle cells was distinguished from that of total hair shaft by levels of more than 20 proteins, the majority of which were prominent keratins. The cuticle also exhibited relatively high levels of epidermal transglutaminase (TGM3), accounting for its observed low degree of protein extraction by denaturants. In addition to providing insight into hair structure, present findings may lead to improvements in differentiating hair from various ethnic origins and offer an approach to extending use of hair in crime scene evidence for distinguishing among individuals.

Narrative summary: This was the first study we found which “revealed considerable variation in [hair proteome] profile among samples from Caucasian, African–American, Kenyan and Korean subjects. Within these ethnic groups, prominent keratin proteins served to distinguish individual profiles. Differences between ethnic groups, less marked, relied to a large extent on levels of keratin associated proteins…. In addition to providing insight into hair structure, present findings may lead to improvements in differentiating hair from various ethnic origins and offer an approach to extending use of hair in crime scene evidence for distinguishing among individuals.” The study mentions previous studies indicating this potential, but this appears to have been the first direct demonstration of ethnic and potential individualizing identifiability.

Reviewer: Sebastian Porsdam Mann, Peter Vilmos Treit
Themes
• Sensitive data/discrimination
o Discrimination
o Sensitivity of data/personal data
• Identifiability/privacy
o Privacy
o Identification/identifiability
o Forensic use
• Standards and quality control
o Public databases: access and data integrity
o Interoperability/inefficiencies

Reviewer: Sebastian Porsdam Mann, Peter Vilmos Treit
Issues
• Discrimination
• Identification/identifiability
• database interoperability/inefficiencies
• Forensic use

ID 48757357: Li (2016)

Li Sujun, Bandeira Nuno and Wang Xiaofeng ; Tang Haixu ;. 2016. "On the privacy risks of sharing clinical proteomics data". AMIA Joint Summits on Translational Science proceedings. AMIA Joint Summits on Translational Science 2016:122-131.
Abstract: Although the privacy issues in human genomic studies are well known, the privacy risks in clinical proteomic data have not been thoroughly studied. As a proof of concept, we reported a comprehensive analysis of the privacy risks in clinical proteomic data. It showed that a small number of peptides carrying the minor alleles (referred to as the minor allelic peptides) at non-synonymous single nucleotide polymorphism (nsSNP) sites can be identified in typical clinical proteomic datasets acquired from the blood/serum samples of individual patient, from which the patient can be identified with high confidence. Our results suggested the presence of significant privacy risks in raw clinical proteomic data. However,

these risks can be mitigated by a straightforward pre-processing step of the raw data that removing a very small fraction (0.1%, 7.14 out of 7,504 spectra on average) of MS/MS spectra identified as the minor allelic peptides, which has little or no impact on the subsequent analysis (and re-use) of these datasets.

Narrative summary: Proof-of-concept study “[showing] that a small number of peptides carrying the minor alleles (referred to as the minor allelic peptides) at non-synonymous single nucleotide polymorphism (nsSNP) sites can be identifed in typical clinical proteomic datasets acquired from the blood/serum samples of individual patient, from which the patient can be identified with high confidence.” The authors therefore warn against privacy risks inherent in raw clinical proteomic data, which they point out is abundant in open source repositories online. They conclude, however, that these risks “can be mitigated by a simple pre-processing of the raw data that removes the MS/MS spectra resulted from the peptides carrying a minor allele at an nsSNP site. We recommended this pre-processing step should be carried out before sharing clinical proteomic datasets in public domain.” The authors also point out that there are other means of identifying individuals from raw clinical proteomics data than nsSNP site identification which they expect to become increasingly important as technology improves.

Reviewer: Sebastian Porsdam Mann, Peter Vilmos Treit
Themes
- Sensitive data/discrimination
  - Discrimination
  - Sensitivity of data/personal data
- Identifiability/privacy
  - Privacy
  - Identification/identifiability
  - Forensic use
- Standards and quality control
  - Public databases: access and data integrity

Reviewer: Sebastian Porsdam Mann
Issues
- Discrimination
- Privacy
- Identification/identifiability
- Public databases: access and data integrity

ID 48757563: Liska (2004)

Liska Adam J. 2004. "The morality of problem selection in proteomics". PROTEOMICS 4(7):1929-1931.
Abstract: Abstract The emerging power of new technologies in proteomics and the biological sciences to alter the human condition demands that scientists hold a new perspective on the social reesponsibilities of their research. Ethical theory can help scientists recognize not only those research projects that are harmful, but also those research paths that can create the greatest improvements in human health on a global scale. Whereas individual choices are important for the direction of scientific research, these choices may have limited social effects if they are not coordinated with larger institutional and inter-institutional structures. The perspective presented here calls for the Human Proteome Organization to recognize the ten most ethically significant proteomes to be characterized, with the hopes of rallying support and directing the research efforts of scientists in the proteomics community toward these goals.

Narrative summary: The article points out that proteomic studies require significant funding, and that the number of potential research topics are therefore limited. This introduces moral choices in study selection. Moreover, the source of funding often influences research topics. The author suggests that individual proteomic scientists, to the extent possible, should attempt to focus their research efforts on topics of broad significance, rather than merely promoting the interests of the funding body. However, the article recognizes that individual efforts may not be enough, and therefore calls for greater institutional and international support. As a specific recommendation, the article calls on HUPO to create a list of the ten most ethically pressing proteomic study topics.

Reviewer: Sebastian Porsdam Mann, Peter Vilmos Treit
Themes

- Conflicting rights and duties
  - Tensions between duties to patients and to science
  - Public v Private (profit)
  - Patient/data subject rights
- Need for normative international guidelines
  - Lack of normative guidelines
  - Trust
  - International collaboration
  - Public awareness/public opinion
  - Social consequences
- Aims and goals of clinical proteomics
  - Funding priorities
  - Aims of research
  - Need for positive ethics beyond regulations
  - Need for discussion within field
- Benefits/Justice
  - Profit/benefit sharing
  - Data sharing
  - Access for low-and-middle-income countries/researchers
  - Solidarity
  - Altruism
  - Fairness
- Regulation

Reviewer: Sebastian Porsdam Mann, Peter Vilmos Treit
Issues

- Profit/benefit sharing
- Lack of international ethical framework/guidance
- Private v Public (Profit, ownership)
- Regulation
- Rights
- Funding priorities/lack of funding
- International collaboration
- Public awareness/public opinion
- Fairness
- Altruism
- Social consequences
- Aims of research

- Need for positive ethics beyond guidelines
- Need for discussion within field

ID 48757561: Martens (2017)

Martens Lennart and Vizcaíno Juan Antonio. 2017. "A Golden Age for Working with Public Proteomics Data". Trends in biochemical sciences 42(5):333-341.
Your ID: 28118949[pmid]
Abstract: Data sharing in mass spectrometry (MS)-based proteomics is becoming a common scientific practice, as is now common in the case of other, more mature 'omics' disciplines like genomics and transcriptomics. We want to highlight that this situation, unprecedented in the field, opens a plethora of opportunities for data scientists. First, we explain in some detail some of the work already achieved, such as systematic reanalysis efforts. We also explain existing applications of public proteomics data, such as proteogenomics and the creation of spectral libraries and spectral archives. Finally, we discuss the main existing challenges and mention the first attempts to combine public proteomics data with other types of omics data sets.

Narrative summary: The authors point out that improvements in data standards across public proteomic databases have massively expanded the utility of raw proteomic data. “In proteomics the number of data types and their corresponding data formats can be overwhelming. ... In a recent review [21], together with other colleagues we established four categories of public proteomics data use: (i) use; (ii) reuse; (iii) reprocess; and (iv) repurpose.” However, the problem of data integrity and quality control is overwhelming: “At present, proteomics resources are assessing the internal consistency of the data submitted (e.g., correspondence between the mass spectra and identification results), detecting clear annotation errors (e.g., related to PTMs), and ensuring an acceptable level of technical and biological metadata… Of course, QC metric calculation at the level of proteomics resources can serve only as a postmortem, as potential issues can no longer be solved at that point. A perfect situation would therefore see QC metrics produced in parallel with data acquisition in the laboratory and subsequently communicated to repositories alongside the data.” Focusing on proteogenomics, the authors identify two major issues: the lack of interoperability and exchange between researchers performing the analyses and those that update genome annotations based on those findings. The other “is the accumulation of false positives, as exemplified in the human proteome draft papers mentioned in Box 3 [Küster and Pandey drafts]. Much more restrictive quality criteria should be established for peptides describing novel genomics events [33]. Moreover, the enlarged sequence search space of typical proteogenomics searches can lead to undesirable ambiguity of identification [40].” The combination of proteogenomics with other omics offers even greater potential. “However, at present, in most cases it is not trivial for researchers to connect data sets that have been generated in multiomics studies… The lack of experimental and technical metadata has been highlighted many times as the main issue for the reuse of biological data, and particularly in proteomics ... In our experience there needs to be a balance between the required amount of metadata and the willingness of researchers to share their data. … Raising the bar in terms of metadata requirements is an achievable goal, as far as proteomics resources have the means to evolve their systems and tools. Unfortunately, the latter can be challenging in the current funding situation as it is often perceived that all issues in this area have been solved. In this context, as a ‘silver lining’, it is important to highlight that the increased adoption of the data standards is key to improving the situation, as much metadata (especially the proteomics-specific metadata) can be extracted automatically from the acquired data files instead of having to be entered manually by the submitters.”

The authors finish on a cautionary note: “In the near future, one challenge that may arise is the existence of limited access to human clinical proteomics data, as is common today for genomics and transcriptomics data sets… Whether access limitations will ultimately apply to clinical proteomics data remains to be seen, but undoubtedly this topic will become an important matter for discussion in the near future…. What privacy and ethics issues will proteomics data raise in the future and what can the field do to adequately prepare for these?”

Reviewer: Sebastian Porsdam Mann, Peter VIlmos Treit
Themes
- Identifiability/privacy
  - Privacy
  - Identification/identifiability
- Standards and quality control
  - Public databases: access and data integrity
  - Need for standards
  - Interoperability/inefficiencies
- Conflicting rights and duties
  - Anonymization and data linkage
- Integration of new technologies and related fields
  - Evolution of technology
  - Complicating factors

Reviewer: Sebastian Porsdam Mann, Peter Vilmos Treit
Issues
- Privacy
- Identification/identifiability
- Public databases: access and data integrity
- Integration with other OMICs and emerging tech: complicating factors
- Need for standards
- database interoperability/inefficiencies

ID 48757354: National (2006)

National Research Council and Stephen A Merrill; Anne-Marie Mazza. 2006. Reaping the Benefits of Genomic and Proteomic Research: Intellectual Property Rights, Innovation, and Public Health. Washington, DC: The National Academies Press.
Abstract: The patenting and licensing of human genetic material and proteins represents an extension of intellectual property (IP) rights to naturally occurring biological material and scientific information, much of it well upstream of drugs and other disease therapies. This report concludes that IP restrictions rarely impose significant burdens on biomedical research, but there are reasons to be apprehensive about their future impact on scientific advances in this area. The report recommends 13 actions that policy-makers, courts, universities, and health and patent officials should take to prevent the increasingly complex web of IP protections from getting in the way of potential breakthroughs in genomic and proteomic research. It endorses the National Institutes of Health guidelines for technology licensing, data sharing, and research material exchanges and says that oversight of compliance should be strengthened. It recommends enactment of a statutory exception from infringement liability for research on a patented invention and raising the bar somewhat to qualify for a patent on upstream research discoveries in biotechnology. With respect to genetic diagnostic tests to detect patient mutations associated with certain diseases, the report urges patent holders to allow others to perform the tests for purposes of verifying the results.

Narrative summary: This was a joint report of the US National Academies focused on the conflicting interested of intellectual property rights, inventors, and public health arising in the context of genomic proteomic advances. It mostly does not distinguish between the two, although in one notable passage it recognizes that “the discipline of proteomics may become an even more commercially important and active patenting arena than DNA because of its closer proximity to disease detection and therapy. Moreover, proteomics may raise novel questions of patent law that must be addressed carefully by a system that for other biological materials has evolved painfully slowly… Terms of access weigh more heavily on those involved in genomics than on those in proteomics, and on those involved in industry-funded research or other commercial activity than on those who are not.”

Reviewer: Sebastian Porsdam Mann, Peter Vilmos Treit
Themes

- Standards and quality control
  - Need for standards
- Conflicting rights and duties
  - Tensions between duties to patients and to science
  - Public v Private (profit)
  - Patient/data subject rights
  - Intellectual property
- Need for normative international guidelines
  - Lack of normative guidelines
  - International collaboration
- Benefits/Justice
  - Profit/benefit sharing
- Regulation

Reviewer: Sebastian Porsdam Mann, Peter Vilmos Treit
Issues

- Tensions between duties to patients and to science
- Profit/benefit sharing
- Lack of international ethical framework/guidance
- Private v Public (Profit, ownership)
- Regulation
- Rights
- Intellectual Property
- Need for standards

ID 48757348: Nestler (2004)

Nestler Grit, Steinert Ralf and Lippert Hans ; Reymond Marc A;. 2004. "Using human samples in proteomics-based drug development: bioethical aspects.". Expert Review of Proteomics 1(1):77-86.
Abstract: Human samples and related medical data are expected to play an elevated role in application-based biomedical proteomics research. Against this framework, some facts should be kept in mind by academic and industrial researchers: international framework conditions on the use of human samples for research purposes are heterogeneous. For example, the value added by the use of human samples for product development is significant and the patient’s personal and property rights may be affected. The body of national laws is growing and these laws are binding; guidelines published by international

organizations should be respected. The most important aspect regards the informed consent of the patient, which is addressed in detail.

Narrative summary: Points out that proteomic studies relies on human samples. Only distinction made is that proteomics will become increasingly important, and so the proteomics community ought to become aware of the general human sample issues. Also calls for bioethics, especially informed consent, and national law to be respected, as well as international guidance (for tissue banks).

Reviewer: Sebastian Porsdam Mann, Peter Vilmos Treit
Themes

- Sensitive data/discrimination
  - Discrimination
  - Sensitivity of data/personal data
- Identifiability/privacy
  - Privacy
  - Identification/identifiability
- Incidental findings/reuse
  - Incidental findings/Secondary analysis
  - Individual relevance of results
- Standards and quality control
  - Need for standards
- Conflicting rights and duties
  - Tensions between duties to patients and to science
  - Patient/data subject rights
  - Intellectual property
- Need for normative international guidelines
  - Lack of normative guidelines
  - Trust
  - International collaboration
  - Social consequences
- Aims and goals of clinical proteomics
  - Need for positive ethics beyond regulations
- Benefits/Justice
  - Profit/benefit sharing
  - Data sharing
  - Solidarity
  - Altruism
  - Fairness
- Regulation
  - Informed Consent

Reviewer: Sebastian Porsdam Mann, Peter Vilmos Treit
Issues

- Discrimination
- Privacy
- Identification/identifiability
- Profit/benefit sharing
- Sensitivity of data/personal data
- Public databases: access and data integrity
- Incidental Findings/Secondary Analysis
- Lack of international ethical framework/guidance
- Trust

- Private v Public (Profit, ownership)
- Informed consent
- Rights
- Intellectual Property
- Need for standards
- Solidarity
- Fairness
- Altruism
- Justice

ID 48757353: Özdemir (2015), Peter Vilmos Treit

Özdemir Vural, Dove Edward S and Gursoy Ulvi Kahraman; Şardaş Semra ; Yıldırım Arif ; Yılmaz Şenay Görücü; Barlas I Ömer; Güngör Kıvanç ; Mete Alper ; Srivastava Sanjeeva ;. 2015. "Personalized medicine beyond genomics: alternative futures in big data-proteomics, environtome and the social proteome.". Journal of Neural Transmission 124(1):25-32.
Abstract: No field in science and medicine today remains untouched by Big Data, and psychiatry is no exception. Proteomics is a Big Data technology and a next generation biomarker, supporting novel system diagnostics and therapeutics in psychiatry. Proteomics technology is, in fact, much older than genomics and dates to the 1970s, well before the launch of the international Human Genome Project. While the genome has long been framed as the master or "elite" executive molecule in cell biology, the proteome by contrast is humble. Yet the proteome is critical for life—it ensures the daily functioning of cells and whole organisms. In short, proteins are the blue-collar workers of biology, the down-to-earth molecules that we cannot live without. Since 2010, proteomics has found renewed meaning and international attention with the launch of the Human Proteome Project and the growing interest in Big Data technologies such as proteomics. This article presents an interdisciplinary technology foresight analysis and conceptualizes the terms "environtome" and "social proteome". We define "environtome" as the entire complement of elements external to the human host, from microbiome, ambient temperature and weather conditions to government innovation policies, stock market dynamics, human values, political power and social norms that collectively shape the human host spatially and temporally. The "social proteome" is the subset of the environtome that influences the transition of proteomics technology to innovative applications in society. The social proteome encompasses, for example, new reimbursement schemes and business innovation models for proteomics diagnostics that depart from the "once-a-life-time" genotypic tests and the anticipated hype attendant to context and time sensitive proteomics tests. Building on the "nesting principle" for governance of complex systems as discussed by Elinor Ostrom, we propose here a 3-tiered organizational architecture for Big Data science such as proteomics. The proposed nested governance structure is comprised of (a) scientists, (b) ethicists, and (c) scholars in the nascent field of "ethics-of-ethics", and aims to cultivate a robust social proteome for personalized medicine. Ostrom often noted that such nested governance designs offer assurance that political power embedded in innovation processes is distributed evenly and is not concentrated disproportionately in a single overbearing stakeholder or person. We agree with this assessment and conclude by underscoring the synergistic value of social and biological proteomes to realize the full potentials of proteomics science for personalized medicine in psychiatry in the present era of Big Data.

Narrative summary: This article focused mainly on societal and sociological aspects of proteomics, but identified several points of ethical interest. Notably, it argued that "[t]he distinctly adaptive capacity of the proteome to capture the dynamic changes in biology and function within an individual means that proteomics test results fluctuate within a given person, either due to disease, drug treatment or baseline physiological within-person variations… Such dynamic biological characteristics of the proteome, however, are likely to translate into certain social consequences such as hype, uncertainty and individuals'

perceptions of their future health risks, in ways distinct from the static genotype-based risk assessment models.” It also noted that “[w]ith the repeated testing necessary to monitor the function of cells and living organisms using proteomics, new diagnostic innovation and business models may become more akin to pharmaceuticals that require repeated prescription fillings.” The authors coin “the social proteome” as a neologism to capture the many environmental impacts on endogenous protein production. This is an important dual insight with considerable, if implicit, normative significance. After pointing to the availability, cost, and affordability concerns surrounding mass-spectrometry as a key “socio-technological factor:… the scientists, institutions and countries with access to mass spectrometry will excel in mapping out the biological proteome. The social proteome, too, will be influenced by the availability of mass spectrometry, for this equipment is expensive, thereby holding substantive potential to shape the professional values, professional competition and cooperation within the proteomics science community. Technologies situated at the epicenter of proteomics such as mass spectrometry can thus shape both the biological and the social proteome; by tracing mass spectrometry as a “socio-material” object, one might better understand the emergence of new socio-technical configurations of proteomics science and its diffusion (or lack of access to proteomics diagnostics in certain regions) around the globe.” The authors provide a table illustrating several sociological concerns and stress that “the scale, quality and multiple application contexts of proteomics technology suggest that the emergence of proteomics science has distinct social, economic, political and medical impacts on society, thus creating a social proteome that is in need of rigorous empirical and normative analyses in the future.” (italics ours). Moreover, the impact is bidirectional: “Conversely, changes in the social proteome, for example, in the ambient social context of a human host, can alter the biological proteome….. As psychiatric illness and therapies are in a state of dynamic interaction between the individual, society and the social systems such as those noted above and in Table 1, it would be beneficial to study in the future the bidirectional impacts of the social proteome on the biological proteome and vice versa.”
Finally, the authors raise the important point that unlike genomics, proteomics has not yet ‘outsourced’ its ELSI concerns to the professional ELSI community. “By situating the context of knowledge co-production and applications of proteomics technology, scientists can help create a “socialized proteome” whereby the social, political, economic and ethical dimensions are surfaced transparently…At this early stage of globalization of proteomics science in the developed and developing world alike, it would be prudent to pause, reflect and learn from the lessons learned from genomics and other emerging technologies in twentieth century. We may want to adopt the view that early engagement with emerging technologies can accrue important social, ethical, economic and policy gains that responsibly benefit many stakeholders. Yet such anticipated gains from new technologies are not automatic…”

Reviewer: Sebastian Porsdam Mann, Peter Vilmos Treit
Themes
- Conflicting rights and duties
  - Public v Private (profit)
- Need for normative international guidelines
  - Lack of normative guidelines
  - Trust
  - International collaboration
  - Public awareness/public opinion
  - Social consequences
- Aims and goals of clinical proteomics
  - Aims of research
  - Need for positive ethics beyond regulations
  - Need for discussion within field
- Benefits/Justice

o Profit/benefit sharing
o Access for low-and-middle-income countries/researchers
o Solidarity
o Altruism
o Fairness
o Authority/power
• Integration of new technologies and related fields
o Evolution of technology
o Complicating factors

Reviewer: Sebastian Porsdam Mann, Peter Vilmos Treit
Issues

- Profit/benefit sharing
- Integration with other OMICs and emerging tech: complicating factors
- Lack of international ethical framework/guidance
- Private v Public (Profit, ownership)
- Access for low-and-middle middle-income countries/researchers
- International collaboration
- Public awareness/public opinion
- Fairness
- Justice
- Social consequences
- Authority/power

ID 48757356: Parker (2016)

Parker Glendon J, Leppert Tami and Anex Deon S; Hilmer Jonathan K; Matsunami Nori ; Baird Lisa ; Stevens Jeffery ; Parsawar Krishna ; Durbin-Johnson Blythe P; Rocke David M; Nelson Chad ; Fairbanks Daniel J; Wilson Andrew S; Rice Robert H; Woodward Scott R; Bothner Brian ; Hart Bradley R; Leppert Mark ;. 2016. "Demonstration of Protein-Based Human Identification Using the Hair Shaft Proteome". PLOS ONE 11(9):e0160653-.
Abstract: Human identification from biological material is largely dependent on the ability to characterize genetic polymorphisms in DNA. Unfortunately, DNA can degrade in the environment, sometimes below the level at which it can be amplified by PCR. Protein however is chemically more robust than DNA and can persist for longer periods. Protein also contains genetic variation in the form of single amino acid polymorphisms. These can be used to infer the status of non-synonymous single nucleotide polymorphism alleles. To demonstrate this, we used mass spectrometry-based shotgun proteomics to characterize hair shaft proteins in 66 European-American subjects. A total of 596 single nucleotide polymorphism alleles were correctly imputed in 32 loci from 22 genes of subjects' DNA and directly validated using Sanger sequencing. Estimates of the probability of resulting individual non-synonymous single nucleotide polymorphism allelic profiles in the European population, using the product rule, resulted in a maximum power of discrimination of 1 in 12,500. Imputed non-synonymous single nucleotide polymorphism profiles from European–American subjects were considerably less frequent in the African population (maximum likelihood ratio = 11,000). The converse was true for hair shafts collected from an additional 10 subjects with African ancestry, where some profiles were more frequent in the African population. Genetically variant peptides were also identified in hair shaft datasets from six archaeological skeletal remains (up to 260 years old). This study demonstrates that quantifiable measures of identity discrimination and biogeographic background can be obtained from detecting genetically variant peptides in hair shaft protein, including hair from bioarchaeological contexts.

Narrative summary: This article points to the frequent use of DNA evidence in forensic as well as bioarchaelogical sciences: “DNA typing has the ability to statistically place individuals at specific locations, to associate them with physical evidence, and to determine biometric and biogeographic genetic information[2–5]. In a bioarchaeological context, ancient DNA allows calculation of biodistance when compared to other samples and existing biogeographic populations[6, 7].“ However, DNA denatures relatively quickly, so the authors investigated the forensic potential for human identification using the hair shaft proteome. Based on nsSNP detection, they were able to derive both identifying and biogeographic information. Specifically, they were able to distinguish between Caucasian, African-American and Kenyan individuals. “When the approach was extended to bioarchaeological hair samples, these individual measures of discrimination and likelihood of biogeographic background, were also obtained.”

Reviewer: Sebastian Porsdam Mann, Peter Vilmos Treit
Themes
- Sensitive data/discrimination
  - Discrimination
  - Sensitivity of data/personal data
- Identifiability/privacy
  - Privacy
  - Identification/identifiability
  - Forensic use

Reviewer: Sebastian Porsdam Mann, Peter Vilmos Treit
Issues
- Discrimination
- Privacy
- Forensic use

ID 48757560: Reymond (2003)

Reymond Marc A, Steinert Ralf and Eder Frank ; Lippert Hans ;. 2003. "Ethical and regulatory issues arising from proteomic research and technology". PROTEOMICS 3(8):1387-1396.
Abstract: Abstract Over the last two decades, medical research has begun to make extensive use of products of human origin in therapeutics, oncology, and most recently, in genetic diseases. This has raised many ethical issues involving patient rights, including issues of consent. Besides informed consent, researchers should address several topics when designing studies using human tissues. Reward for the patient should be kept minimal. Sample transfer should be organized along non-profit lines, at least in Europe. Sampling procedures should be at no risk for human volunteers, and at minimal risk for patients. Biosafety aspects should be addressed, in particular when international collaborations are intended or when collaboration is existing between academia and industry. Regulations on importation and exportation of human tissues should be observed. Data acquisition and storage should be addressed in accordance with national data protection regulations, in particular when using computerized databases. If follow-up information is to be taken, the authorization for such information should be requested. The right for patient?s information (or for no information) on the research results should also be addressed. The issues of validation and patenting should be also solved, usually by informing the patient that he/she will have no commercial rights on potential research results. The patient should be told if the samples are transferred to another research laboratory or private company. Samples and related data should be destroyed on request at any time point during the course of the study. If possible, traceability of the donor should be ensured.

Narrative summary: The article notes growing concerns regarding the use of human tissues in biobanks or biorepositories, especially related to ownership and profit. It notes that ethical and regulatory guidance is vague and varies widely between countries. There are no international guidelines. The article suggests that these should be based on bioethical principles and international human rights law. It then delves into specific distinguishing features of proteomics. Firstly, proteomics allows for the investigation of environmental effects over time – it is dynamic while genomics is static. "As a logical consequence, they often require repeated analyses over the course of disease. Of course, anonymization of probes is not directly compatible with such follow-up studies." It suggests that this problem "can be circumvented by contracting a third party for anonymization and follow-up tasks, so that the biological information never comes in to contact with the patient's identity." It then points to two further distinguishing features: the need for large quantities of blood in proteomics studies, and its inability to enhance genomic information. Although these features remain relevant, the situation is reversed in 2020: very small quantities of blood are required, and much enhancement of genomic information is possible. The paper then suggests a series of recommendations essentially based on medical ethics codes. Of these, the most novel and important relates to the issue of intellectual property, proteomics, and the sharing of benefits. Finally, the author stressed the importance of informed consent with several options for levels of data sharing and privacy risks.

Reviewer: Sebastian Porsdam Mann, Peter Vilmos Treit
Themes
- Sensitive data/discrimination
  - Discrimination
  - Sensitivity of data/personal data
- Identifiability/privacy
  - Privacy
  - Identification/identifiability
- Incidental findings/reuse
  - Individual relevance of results
- Standards and quality control
  - Public databases: access and data integrity
  - Need for standards
- Conflicting rights and duties
  - Tensions between duties to patients and to science
  - Anonymization and data linkage
  - Economic incentives for data sharing/undue inducement
  - Public v Private (profit)
  - Patient/data subject rights
  - Intellectual property
- Need for normative international guidelines
  - Lack of normative guidelines
  - International collaboration
- Aims and goals of clinical proteomics
  - Need for positive ethics beyond regulations
  - Need for discussion within field
- Benefits/Justice
  - Profit/benefit sharing
  - Data sharing
- Regulation
  - Informed Consent

Reviewer: Sebastian Porsdam Mann, Peter Vilmos Treit
Issues
- Privacy
- Identification/identifiability
- Profit/benefit sharing
- Data sharing
- Lack of international ethical framework/guidance
- Economic incentives for data sharing
- Private v Public (Profit, ownership)
- Regulation
- Informed consent
- Rights
- Intellectual Property
- Need for standards
- International collaboration

ID 48757355: Twyman (2012)

Twyman R M and Chadwick Ruth. 2012. Proteomics. In , edited by , 642-649. San Diego: Academic Press.
Abstract: Human samples and large derivative data sets are necessary for biomedical proteomics research and the development of new drugs using proteomics data. The likelihood that such data will be used in a predictive manner raises ethical concerns, principally the impact on privacy, which means it is necessary to balance the needs of medical research with the needs and rights of the patient. Another important ethical challenge is the issue of property rights (and intellectual property rights) arising from the collection, storage, and dissemination of biological samples and the proteomic data generated from them. There is no consistent international regulatory framework in place. The outstanding ethical issues are currently addressed by seeking the informed consent of the donor, but it is not clear that current forms of informed consent provide donors and patients with sufficient information and suitable choices.

Narrative summary: This was the first article we found that distinguished clearly between genomics and proteomics and focused on the ethical issues specific to proteomics. The author notes that “Proteomics provides a much more robust and representative picture of the functioning cell than do other forms of large-scale biology, such as genome sequencing or the global analysis of gene expression; therefore, the potential ethical risks associated with sample and data misuse are greater.” He notes the importance of identifiability: “[t]he ethical issues raised by the separation of proteins reflect the fact that such methods could, and indeed do, provide molecular fingerprints that can be used to identify individuals, ethnic groups, and, in a clinical setting, groups of individuals with or susceptible to specific diseases,” and later makes clear the link between identification and the possibility “that protein fingerprints could be used to discriminate against individuals and groups... Where such data are used solely for the clinical benefit of the patient, this would be acceptable. The danger lies in any possibility that proteomic analysis would be used to predict complex medical or even psychological outcomes and deny individuals with specific proteomic profiles insurance or employment or cause invasions of privacy.”
The author notes that these issues are similar to their genomic counterparts, but that the proteomics context renders them “more imperative ethical challenges compared to geno¬mics, with the greater imperative resulting from the greater resolution, diversity, and dynamism of proteomics data compared to DNA sequences, and the wider range of samples that can be used for proteomic analysis compared to DNA. For example, proteomics can be applied to ancient samples in which DNA has degraded beyond use, to fixed tissue specimens, and to body tissues and fluids that lack DNA, such as serum, red blood cells, and spinal fluid. This means that proteomics may provide precise molecular data in cases in

which DNA evidence is impossible to secure." He also points to another significant difference: "DNA resources are potentially infinite, whereas proteomic resources are not…However, any data derived from the samples can be used repetitively, either for comparison with a different data set or, where the raw data are stored, for mining to address any number of different questions." He therefore underscores the calls for broader or more dynamic consent options, giving participants more information and further options at the point of consent to reflect the greater possibilities of reuse and the lesser foreseeability of these uses. The article also calls for high levels of data security and anonymization wherever possible.

Most importantly, the article notes that "[t]he regulatory oversight for the use of human material for proteomic research differs in different countries and is evolving, but harmonization is being developed in the form of the Universal Declaration on the Human Genome and Human Rights proposed by the United Nations in 1998, which focuses on protection of human health and safety, human (especially patient) rights, commercial rights, intellectual property rights, and international regulations…..Another important ethical challenge is the issue of property rights (and intellectual property rights) arising from the collection, storage, and dissemination of biological samples and the proteomic data generated from them. A harmonized international regulatory framework would be beneficial in this context, but no consistent framework is in place. The outstanding ethical issues are currently addressed by seeking the informed consent of the donor, but it is imperative that informed consent provides donors and patients with sufficient information and suitable choices."

Reviewer: Sebastian Porsdam Mann, Peter Vilmos Treit

Themes

- Sensitive data/discrimination
  - Discrimination
  - Sensitivity of data/personal data
- Identifiability/privacy
  - Privacy
  - Identification/identifiability
  - Forensic use
  - Third party identifiability
- Incidental findings/reuse
  - Incidental findings/Secondary analysis
  - Uncertainty
  - Individual relevance of results
- Standards and quality control
  - Public databases: access and data integrity
  - Need for standards
  - Interoperability/inefficiencies
- Conflicting rights and duties
  - Tensions between duties to patients and to science
  - Anonymization and data linkage
  - Public v Private (profit)
  - Patient/data subject rights
  - Intellectual property
- Need for normative international guidelines
  - Lack of normative guidelines
  - International collaboration
  - Public awareness/public opinion
- Aims and goals of clinical proteomics
  - Need for positive ethics beyond regulations
  - Need for discussion within field

- Benefits/Justice
  - Data sharing
- Regulation
  - Informed Consent

Reviewer: Sebastian Porsdam Mann, Peter Vilmos Treit
Issues

- Discrimination
- Privacy
- Identification/identifiability
- Data sharing
- Sensitivity of data/personal data
- Public databases: access and data integrity
- Lack of international ethical framework/guidance
- Private v Public (Profit, ownership)
- Informed consent
- Rights
- database interoperability/inefficiencies
- Third party identifiability

through sample processing and handling (technical effects). The inherent variation present within the samples must also be taken into account. In this review, we examine the impact of these factors and issues to be faced when banking samples with a particular focus on sources of pre-analytical variation, which must be rigorously controlled and recorded. It is encouraging that several initiatives are now addressing such key issues and these are also discussed.

Narrative summary: In this proteomics-specific review, the effects of pre-analytical steps and general logistics of storing samples in biobanks are explored. From an ethical and legal perspective, the paper highlights the difficulty in that samples might be used for a different purpose as originally intended, as well as different national and multinational codes of conduct that protect both the donor and researcher wishing to analyze the sample. Furthermore, the paper highlights potential steps to ensure secure sample and data collection (clinical metadata, demographic and storage data), storage and access. Measures taken here should allow for avoiding biases, adherence to good clinical practice and compatibility with clinical trials. The paper’s main focus is on pre-analytical variation, and the plethora of ways methods relating to samples need to be standardised in order to adhere to clinical standards. These areas include steps technical steps such sample collection, storage, processing and processing, parameters that vary widely for the studied material (biofluid / tissue). The paper also highlights the biological variation as something to keep in mind, and that this is an active area of research where standardisation of sample protocols and solid systematic reviews are thus far lacking.

Reviewer: Sebastian Porsdam Mann
Themes

- Standards and quality control
  - Public databases: access and data integrity
  - Need for standards
  - Interoperability/inefficiencies
- Need for normative international guidelines

o Lack of normative guidelines
o International collaboration
• Regulation

Reviewer: Sebastian Porsdam Mann, Peter Vilmos Treit
Issues
• Public databases: access and data integrity
• Lack of international ethical framework/guidance
• Regulation
• Need for standards

ID 48757358: Laatsch (2014)

Laatsch Chelsea N, Durbin-Johnson Blythe P; Rocke David M; Mukwana Sophie and Newland Abby B; Flagler Michael J; Davis Michael G; Eigenheer Richard A; Phinney Brett S; Rice Robert H;. 2014. "Human hair shaft proteomic profiling: individual differences, site specificity and cuticle analysis". PeerJ 2:e506-e506.
Abstract: Hair from different individuals can be distinguished by physical properties. Although some data exist on other species, examination of the individual molecular differences within the human hair shaft has not been thoroughly investigated. Shotgun proteomic analysis revealed considerable variation in profile among samples from Caucasian, African-American, Kenyan and Korean subjects. Within these ethnic groups, prominent keratin proteins served to distinguish individual profiles. Differences between ethnic groups, less marked, relied to a large extent on levels of keratin associated proteins. In samples from Caucasian subjects, hair shafts from axillary, beard, pubic and scalp regions exhibited distinguishable profiles, with the last being most different from the others. Finally, the profile of isolated hair cuticle cells was distinguished from that of total hair shaft by levels of more than 20 proteins, the majority of which were prominent keratins. The cuticle also exhibited relatively high levels of epidermal transglutaminase (TGM3), accounting for its observed low degree of protein extraction by denaturants. In addition to providing insight into hair structure, present findings may lead to improvements in differentiating hair from various ethnic origins and offer an approach to extending use of hair in crime scene evidence for distinguishing among individuals.

Narrative summary: This was the first study we found which "revealed considerable variation in [hair proteome] profile among samples from Caucasian, African–American, Kenyan and Korean subjects. Within these ethnic groups, prominent keratin proteins served to distinguish individual profiles. Differences between ethnic groups, less marked, relied to a large extent on levels of keratin associated proteins…. In addition to providing insight into hair structure, present findings may lead to improvements in differentiating hair from various ethnic origins and offer an approach to extending use of hair in crime scene evidence for distinguishing among individuals." The study mentions previous studies indicating this potential, but this appears to have been the first direct demonstration of ethnic and potential individualizing identifiability.

Reviewer: Sebastian Porsdam Mann, Peter Vilmos Treit
Themes
• Sensitive data/discrimination
o Discrimination
o Sensitivity of data/personal data
• Identifiability/privacy
o Privacy
o Identification/identifiability

o Forensic use
• Standards and quality control
o Public databases: access and data integrity
o Interoperability/inefficiencies

Reviewer: Sebastian Porsdam Mann, Peter Vilmos Treit
Issues
• Discrimination
• Identification/identifiability
• database interoperability/inefficiencies
• Forensic use

ID 48757357: Li (2016)

Li Sujun, Bandeira Nuno and Wang Xiaofeng ; Tang Haixu ;. 2016. "On the privacy risks of sharing clinical proteomics data". AMIA Joint Summits on Translational Science proceedings. AMIA Joint Summits on Translational Science 2016:122-131.
Abstract: Although the privacy issues in human genomic studies are well known, the privacy risks in clinical proteomic data have not been thoroughly studied. As a proof of concept, we reported a comprehensive analysis of the privacy risks in clinical proteomic data. It showed that a small number of peptides carrying the minor alleles (referred to as the minor allelic peptides) at non-synonymous single nucleotide polymorphism (nsSNP) sites can be identified in typical clinical proteomic datasets acquired from the blood/serum samples of individual patient, from which the patient can be identified with high confidence. Our results suggested the presence of significant privacy risks in raw clinical proteomic data. However, these risks can be mitigated by a straightforward pre-processing step of the raw data that removing a very small fraction (0.1%, 7.14 out of 7,504 spectra on average) of MS/MS spectra identified as the minor allelic peptides, which has little or no impact on the subsequent analysis (and re-use) of these datasets.

Narrative summary: Proof-of-concept study “[showing] that a small number of peptides carrying the minor alleles (referred to as the minor allelic peptides) at non-synonymous single nucleotide polymorphism (nsSNP) sites can be identifed in typical clinical proteomic datasets acquired from the blood/serum samples of individual patient, from which the patient can be identified with high confidence.” The authors therefore warn against privacy risks inherent in raw clinical proteomic data, which they point out is abundant in open source repositories online. They conclude, however, that these risks “can be mitigated by a simple pre-processing of the raw data that removes the MS/MS spectra resulted from the peptides carrying a minor allele at an nsSNP site. We recommended this pre-processing step should be carried out before sharing clinical proteomic datasets in public domain.” The authors also point out that there are other means of identifying individuals from raw clinical proteomics data than nsSNP site identification which they expect to become increasingly important as technology improves.

Reviewer: Sebastian Porsdam Mann, Peter Vilmos Treit
Themes
• Sensitive data/discrimination
o Discrimination
o Sensitivity of data/personal data
• Identifiability/privacy
o Privacy
o Identification/identifiability
o Forensic use

- Standards and quality control
  - Public databases: access and data integrity

Reviewer: Sebastian Porsdam Mann, Peter Vilmos Treit
Issues
- Discrimination
- Privacy
- Identification/identifiability
- Public databases: access and data integrity

ID 48757563: Liska (2004)

Liska Adam J. 2004. "The morality of problem selection in proteomics". PROTEOMICS 4(7):1929-1931.
Abstract: Abstract The emerging power of new technologies in proteomics and the biological sciences to alter the human condition demands that scientists hold a new perspective on the social reesponsibilities of their research. Ethical theory can help scientists recognize not only those research projects that are harmful, but also those research paths that can create the greatest improvements in human health on a global scale. Whereas individual choices are important for the direction of scientific research, these choices may have limited social effects if they are not coordinated with larger institutional and inter-institutional structures. The perspective presented here calls for the Human Proteome Organization to recognize the ten most ethically significant proteomes to be characterized, with the hopes of rallying support and directing the research efforts of scientists in the proteomics community toward these goals.

Narrative summary: The article points out that proteomic studies require significant funding, and that the number of potential research topics are therefore limited. This introduces moral choices in study selection. Moreover, the source of funding often influences research topics. The author suggests that individual proteomic scientists, to the extent possible, should attempt to focus their research efforts on topics of broad significance, rather than merely promoting the interests of the funding body. However, the article recognizes that individual efforts may not be enough, and therefore calls for greater institutional and international support. As a specific recommendation, the article calls on HUPO to create a list of the ten most ethically pressing proteomic study topics.

Reviewer: Sebastian Porsdam Mann, Peter Vilmos Treit
Themes
- Conflicting rights and duties
  - Tensions between duties to patients and to science
  - Public v Private (profit)
  - Patient/data subject rights
- Need for normative international guidelines
  - Lack of normative guidelines
  - Trust
  - International collaboration
  - Public awareness/public opinion
  - Social consequences
- Aims and goals of clinical proteomics
  - Funding priorities
  - Aims of research

o Need for positive ethics beyond regulations
o Need for discussion within field
• Benefits/Justice
o Profit/benefit sharing
o Data sharing
o Access for low-and-middle-income countries/researchers
o Solidarity
o Altruism
o Fairness
• Regulation

Reviewer: Sebastian Porsdam Mann, Peter Vilmos Treit
Issues
• Profit/benefit sharing
• Lack of international ethical framework/guidance
• Private v Public (Profit, ownership)
• Regulation
• Rights
• Funding priorities/lack of funding
• International collaboration
• Public awareness/public opinion
• Fairness
• Altruism
• Social consequences
• Aims of research
• Need for positive ethics beyond guidelines
• Need for discussion within field

ID 48757561: Martens (2017)

Martens Lennart and Vizcaíno Juan Antonio. 2017. "A Golden Age for Working with Public Proteomics Data". Trends in biochemical sciences 42(5):333-341.
Your ID: 28118949[pmid]
Abstract: Data sharing in mass spectrometry (MS)-based proteomics is becoming a common scientific practice, as is now common in the case of other, more mature 'omics' disciplines like genomics and transcriptomics. We want to highlight that this situation, unprecedented in the field, opens a plethora of opportunities for data scientists. First, we explain in some detail some of the work already achieved, such as systematic reanalysis efforts. We also explain existing applications of public proteomics data, such as proteogenomics and the creation of spectral libraries and spectral archives. Finally, we discuss the main existing challenges and mention the first attempts to combine public proteomics data with other types of omics data sets.

Narrative summary: The authors point out that improvements in data standards across public proteomic databases have massively expanded the utility of raw proteomic data. “In proteomics the number of data types and their corresponding data formats can be overwhelming. ... In a recent review [21], together with other colleagues we established four categories of public proteomics data use: (i) use; (ii) reuse; (iii) reprocess; and (iv) repurpose.” However, the problem of data integrity and quality control is overwhelming: “At present, proteomics resources are assessing the internal consistency of the data submitted (e.g., correspondence between the mass spectra and identification results), detecting clear annotation errors (e.g., related to PTMs), and ensuring an acceptable level of technical and biological metadata… Of course, QC metric calculation at the level of proteomics resources

can serve only as a postmortem, as potential issues can no longer be solved at that point. A perfect situation would therefore see QC metrics produced in parallel with data acquisition in the laboratory and subsequently communicated to repositories alongside the data."
Focusing on proteogenomics, the authors identify two major issues: the lack of interoperability and exchange between researchers performing the analyses and those that update genome annotations based on those findings. The other "is the accumulation of false positives, as exemplified in the human proteome draft papers mentioned in Box 3 [Küster and Pandey drafts]. Much more restrictive quality criteria should be established for peptides describing novel genomics events [33]. Moreover, the enlarged sequence search space of typical proteogenomics searches can lead to undesirable ambiguity of identification [40]."
The combination of proteogenomics with other omics offers even greater potential. "However, at present, in most cases it is not trivial for researchers to connect data sets that have been generated in multiomics studies… The lack of experimental and technical metadata has been highlighted many times as the main issue for the reuse of biological data, and particularly in proteomics ... In our experience there needs to be a balance between the required amount of metadata and the willingness of researchers to share their data. … Raising the bar in terms of metadata requirements is an achievable goal, as far as proteomics resources have the means to evolve their systems and tools. Unfortunately, the latter can be challenging in the current funding situation as it is often perceived that all issues in this area have been solved. In this context, as a 'silver lining', it is important to highlight that the increased adoption of the data standards is key to improving the situation, as much metadata (especially the proteomics-specific metadata) can be extracted automatically from the acquired data files instead of having to be entered manually by the submitters."

The authors finish on a cautionary note: "In the near future, one challenge that may arise is the existence of limited access to human clinical proteomics data, as is common today for genomics and transcriptomics data sets… Whether access limitations will ultimately apply to clinical proteomics data remains to be seen, but undoubtedly this topic will become an important matter for discussion in the near future…. What privacy and ethics issues will proteomics data raise in the future and what can the field do to adequately prepare for these?"

Reviewer: Sebastian Porsdam Mann, Peter Vilmos Treit
Themes

- Identifiability/privacy
  - Privacy
  - Identification/identifiability
- Standards and quality control
  - Public databases: access and data integrity
  - Need for standards
  - Interoperability/inefficiencies
- Conflicting rights and duties
  - Anonymization and data linkage
- Integration of new technologies and related fields
  - Evolution of technology
  - Complicating factors

Reviewer: Sebastian Porsdam Mann, Peter Vilmos Treit

Issues

- Privacy

- Identification/identifiability
- Public databases: access and data integrity
- Integration with other OMICs and emerging tech: complicating factors
- Need for standards
- database interoperability/inefficiencies

ID 48757354: National (2006)

National Research Council and Stephen A Merrill; Anne-Marie Mazza. 2006. Reaping the Benefits of Genomic and Proteomic Research: Intellectual Property Rights, Innovation, and Public Health. Washington, DC: The National Academies Press.
Abstract: The patenting and licensing of human genetic material and proteins represents an extension of intellectual property (IP) rights to naturally occurring biological material and scientific information, much of it well upstream of drugs and other disease therapies. This report concludes that IP restrictions rarely impose significant burdens on biomedical research, but there are reasons to be apprehensive about their future impact on scientific advances in this area. The report recommends 13 actions that policy-makers, courts, universities, and health and patent officials should take to prevent the increasingly complex web of IP protections from getting in the way of potential breakthroughs in genomic and proteomic research. It endorses the National Institutes of Health guidelines for technology licensing, data sharing, and research material exchanges and says that oversight of compliance should be strengthened. It recommends enactment of a statutory exception from infringement liability for research on a patented invention and raising the bar somewhat to qualify for a patent on upstream research discoveries in biotechnology. With respect to genetic diagnostic tests to detect patient mutations associated with certain diseases, the report urges patent holders to allow others to perform the tests for purposes of verifying the results.

Narrative summary: This was a joint report of the US National Academies focused on the conflicting interested of intellectual property rights, inventors, and public health arising in the context of genomic proteomic advances. It mostly does not distinguish between the two, although in one notable passage it recognizes that “the discipline of proteomics may become an even more commercially important and active patenting arena than DNA because of its closer proximity to disease detection and therapy. Moreover, proteomics may raise novel questions of patent law that must be addressed carefully by a system that for other biological materials has evolved painfully slowly… Terms of access weigh more heavily on those involved in genomics than on those in proteomics, and on those involved in industry-funded research or other commercial activity than on those who are not.”

Reviewer: Sebastian Porsdam Mann, Peter Vilmos Treit
Themes
- Standards and quality control
    - Need for standards
- Conflicting rights and duties
    - Tensions between duties to patients and to science
    - Public v Private (profit)
    - Patient/data subject rights
    - Intellectual property
- Need for normative international guidelines
    - Lack of normative guidelines
    - International collaboration
- Benefits/Justice
    - Profit/benefit sharing

- Regulation

Reviewer: Sebastian Porsdam Mann, Peter Vilmos Treit
Issues

- Tensions between duties to patients and to science
- Profit/benefit sharing
- Lack of international ethical framework/guidance
- Private v Public (Profit, ownership)
- Regulation
- Rights
- Intellectual Property
- Need for standards

ID 48757348: Nestler (2004)

Nestler Grit, Steinert Ralf and Lippert Hans ; Reymond Marc A;. 2004. "Using human samples in proteomics-based drug development: bioethical aspects.". Expert Review of Proteomics 1(1):77-86.
Abstract: Human samples and related medical data are expected to play an elevated role in application-based biomedical proteomics research. Against this framework, some facts should be kept in mind by academic and industrial researchers: international framework conditions on the use of human samples for research purposes are heterogeneous. For example, the value added by the use of human samples for product development is significant and the patient's personal and property rights may be affected. The body of national laws is growing and these laws are binding; guidelines published by international organizations should be respected. The most important aspect regards the informed consent of the patient, which is addressed in detail.

Narrative summary: Points out that proteomic studies relies on human samples. Only distinction made is that proteomics will become increasingly important, and so the proteomics community ought to become aware of the general human sample issues. Also calls for bioethics, especially informed consent, and national law to be respected, as well as international guidance (for tissue banks).

Reviewer: Sebastian Porsdam Mann, Peter Vilmos Treit
Themes

- Sensitive data/discrimination
  - Discrimination
  - Sensitivity of data/personal data
- Identifiability/privacy
  - Privacy
  - Identification/identifiability
- Incidental findings/reuse
  - Incidental findings/Secondary analysis
  - Individual relevance of results
- Standards and quality control
  - Need for standards
- Conflicting rights and duties
  - Tensions between duties to patients and to science
  - Patient/data subject rights
  - Intellectual property
- Need for normative international guidelines

o Lack of normative guidelines
o Trust
o International collaboration
o Social consequences
• Aims and goals of clinical proteomics
o Need for positive ethics beyond regulations
• Benefits/Justice
o Profit/benefit sharing
o Data sharing
o Solidarity
o Altruism
o Fairness
• Regulation
o Informed Consent

Reviewer: Sebastian Porsdam Mann, Peter Vilmos Treit
Issues
• Discrimination
• Privacy
• Identification/identifiability
• Profit/benefit sharing
• Sensitivity of data/personal data
• Public databases: access and data integrity
• Incidental Findings/Secondary Analysis
• Lack of international ethical framework/guidance
• Trust
• Private v Public (Profit, ownership)
• Informed consent
• Rights
• Intellectual Property
• Need for standards
• Solidarity
• Fairness
• Altruism
• Justice

ID 48757353: Özdemir (2015)

Özdemir Vural, Dove Edward S and Gursoy Ulvi Kahraman; Şardaş Semra ; Yıldırım Arif ; Yılmaz Şenay Görücü; Barlas I Ömer; Güngör Kıvanç ; Mete Alper ; Srivastava Sanjeeva ;. 2015. "Personalized medicine beyond genomics: alternative futures in big data-proteomics, environtome and the social proteome.". Journal of Neural Transmission 124(1):25-32.
Abstract: No field in science and medicine today remains untouched by Big Data, and psychiatry is no exception. Proteomics is a Big Data technology and a next generation biomarker, supporting novel system diagnostics and therapeutics in psychiatry. Proteomics technology is, in fact, much older than genomics and dates to the 1970s, well before the launch of the international Human Genome Project. While the genome has long been framed as the master or “elite” executive molecule in cell biology, the proteome by contrast is humble. Yet the proteome is critical for life—it ensures the daily functioning of cells and whole organisms. In short, proteins are the blue-collar workers of biology, the down-to-earth molecules that we cannot live without. Since 2010, proteomics has found renewed meaning and international attention with the launch of the Human Proteome Project and the growing interest in Big Data technologies such as proteomics. This article presents an interdisciplinary technology foresight analysis and conceptualizes the terms “environtome”

and “social proteome”. We define “environtome” as the entire complement of elements external to the human host, from microbiome, ambient temperature and weather conditions to government innovation policies, stock market dynamics, human values, political power and social norms that collectively shape the human host spatially and temporally. The “social proteome” is the subset of the environtome that influences the transition of proteomics technology to innovative applications in society. The social proteome encompasses, for example, new reimbursement schemes and business innovation models for proteomics diagnostics that depart from the “once-a-life-time” genotypic tests and the anticipated hype attendant to context and time sensitive proteomics tests. Building on the “nesting principle” for governance of complex systems as discussed by Elinor Ostrom, we propose here a 3-tiered organizational architecture for Big Data science such as proteomics. The proposed nested governance structure is comprised of (a) scientists, (b) ethicists, and (c) scholars in the nascent field of “ethics-of-ethics”, and aims to cultivate a robust social proteome for personalized medicine. Ostrom often noted that such nested governance designs offer assurance that political power embedded in innovation processes is distributed evenly and is not concentrated disproportionately in a single overbearing stakeholder or person. We agree with this assessment and conclude by underscoring the synergistic value of social and biological proteomes to realize the full potentials of proteomics science for personalized medicine in psychiatry in the present era of Big Data.

Narrative summary: This article focused mainly on societal and sociological aspects of proteomics, but identified several points of ethical interest. Notably, it argued that “[t]he distinctly adaptive capacity of the proteome to capture the dynamic changes in biology and function within an individual means that proteomics test results fluctuate within a given person, either due to disease, drug treatment or baseline physiological within-person variations… Such dynamic biological characteristics of the proteome, however, are likely to translate into certain social consequences such as hype, uncertainty and individuals’ perceptions of their future health risks, in ways distinct from the static genotype-based risk assessment models.” It also noted that “[w]ith the repeated testing necessary to monitor the function of cells and living organisms using proteomics, new diagnostic innovation and business models may become more akin to pharmaceuticals that require repeated prescription fillings.” The authors coin “the social proteome” as a neologism to capture the many environmental impacts on endogenous protein production. This is an important dual insight with considerable, if implicit, normative significance. After pointing to the availability, cost, and affordability concerns surrounding mass-spectrometry as a key “socio-technological factor:… the scientists, institutions and countries with access to mass spectrometry will excel in mapping out the biological proteome. The social proteome, too, will be influenced by the availability of mass spectrometry, for this equipment is expensive, thereby holding substantive potential to shape the professional values, professional competition and cooperation within the proteomics science community. Technologies situated at the epicenter of proteomics such as mass spectrometry can thus shape both the biological and the social proteome; by tracing mass spectrometry as a “socio-material” object, one might better understand the emergence of new socio-technical configurations of proteomics science and its diffusion (or lack of access to proteomics diagnostics in certain regions) around the globe.” The authors provide a table illustrating several sociological concerns and stress that “the scale, quality and multiple application contexts of proteomics technology suggest that the emergence of proteomics science has distinct social, economic, political and medical impacts on society, thus creating a social proteome that is in need of rigorous empirical and normative analyses in the future.” (italics ours). Moreover, the impact is bidirectional: “Conversely, changes in the social proteome, for example, in the ambient social context of a human host, can alter the biological proteome….. As psychiatric illness and therapies are in a state of dynamic interaction between the individual, society and the social systems such as those noted above and in Table 1, it would be beneficial to study in the future the bidirectional impacts of the social proteome on the biological proteome and vice versa.”

Finally, the authors raise the important point that unlike genomics, proteomics has not yet 'outsourced' its ELSI concerns to the professional ELSI community. "By situating the context of knowledge co-production and applications of proteomics technology, scientists can help create a "socialized proteome" whereby the social, political, economic and ethical dimensions are surfaced transparently…At this early stage of globalization of proteomics science in the developed and developing world alike, it would be prudent to pause, reflect and learn from the lessons learned from genomics and other emerging technologies in twentieth century. We may want to adopt the view that early engagement with emerging technologies can accrue important social, ethical, economic and policy gains that responsibly benefit many stakeholders. Yet such anticipated gains from new technologies are not automatic…"

Reviewer: Sebastian Porsdam Mann, Peter Vilmos Treit
Themes

- Conflicting rights and duties
  - Public v Private (profit)
- Need for normative international guidelines
  - Lack of normative guidelines
  - Trust
  - International collaboration
  - Public awareness/public opinion
  - Social consequences
- Aims and goals of clinical proteomics
  - Aims of research
  - Need for positive ethics beyond regulations
  - Need for discussion within field
- Benefits/Justice
  - Profit/benefit sharing
  - Access for low-and-middle-income countries/researchers
  - Solidarity
  - Altruism
  - Fairness
  - Authority/power
- Integration of new technologies and related fields
  - Evolution of technology
  - Complicating factors

Reviewer: Sebastian Porsdam Mann, Peter Vilmos Treit
Issues

- Profit/benefit sharing
- Integration with other OMICs and emerging tech: complicating factors
- Lack of international ethical framework/guidance
- Private v Public (Profit, ownership)
- Access for low-and-middle middle-income countries/researchers
- International collaboration
- Public awareness/public opinion
- Fairness
- Justice
- Social consequences
- Authority/power

ID 48757356: Parker (2016)

Parker Glendon J, Leppert Tami and Anex Deon S; Hilmer Jonathan K; Matsunami Nori ; Baird Lisa ; Stevens Jeffery ; Parsawar Krishna ; Durbin-Johnson Blythe P; Rocke David M; Nelson Chad ; Fairbanks Daniel J; Wilson Andrew S; Rice Robert H; Woodward Scott R; Bothner Brian ; Hart Bradley R; Leppert Mark ;. 2016. "Demonstration of Protein-Based Human Identification Using the Hair Shaft Proteome". PLOS ONE 11(9):e0160653-.
Abstract: Human identification from biological material is largely dependent on the ability to characterize genetic polymorphisms in DNA. Unfortunately, DNA can degrade in the environment, sometimes below the level at which it can be amplified by PCR. Protein however is chemically more robust than DNA and can persist for longer periods. Protein also contains genetic variation in the form of single amino acid polymorphisms. These can be used to infer the status of non-synonymous single nucleotide polymorphism alleles. To demonstrate this, we used mass spectrometry-based shotgun proteomics to characterize hair shaft proteins in 66 European-American subjects. A total of 596 single nucleotide polymorphism alleles were correctly imputed in 32 loci from 22 genes of subjects' DNA and directly validated using Sanger sequencing. Estimates of the probability of resulting individual non-synonymous single nucleotide polymorphism allelic profiles in the European population, using the product rule, resulted in a maximum power of discrimination of 1 in 12,500. Imputed non-synonymous single nucleotide polymorphism profiles from European–American subjects were considerably less frequent in the African population (maximum likelihood ratio = 11,000). The converse was true for hair shafts collected from an additional 10 subjects with African ancestry, where some profiles were more frequent in the African population. Genetically variant peptides were also identified in hair shaft datasets from six archaeological skeletal remains (up to 260 years old). This study demonstrates that quantifiable measures of identity discrimination and biogeographic background can be obtained from detecting genetically variant peptides in hair shaft protein, including hair from bioarchaeological contexts.

Narrative summary: This article points to the frequent use of DNA evidence in forensic as well as bioarchaelogical sciences: “DNA typing has the ability to statistically place individuals at specific locations, to associate them with physical evidence, and to determine biometric and biogeographic genetic information[2–5]. In a bioarchaeological context, ancient DNA allows calculation of biodistance when compared to other samples and existing biogeographic populations[6, 7].“ However, DNA denatures relatively quickly, so the authors investigated the forensic potential for human identification using the hair shaft proteome. Based on nsSNP detection, they were able to derive both identifying and biogeographic information. Specifically, they were able to distinguish between Caucasian, African-American and Kenyan individuals. “When the approach was extended to bioarchaeological hair samples, these individual measures of discrimination and likelihood of biogeographic background, were also obtained.”

Reviewer: Sebastian Porsdam Mann, Peter Vilmos Treit

Themes
- Sensitive data/discrimination
  - Discrimination
  - Sensitivity of data/personal data
- Identifiability/privacy
  - Privacy
  - Identification/identifiability
  - Forensic use

Reviewer: Sebastian Porsdam Mann, Peter Vilmos Treit

Issues
- Discrimination
- Privacy
- Forensic use

ID 48757560: Reymond (2003)

Reymond Marc A, Steinert Ralf and Eder Frank ; Lippert Hans ;. 2003. "Ethical and regulatory issues arising from proteomic research and technology". PROTEOMICS 3(8):1387-1396.
Abstract: Abstract Over the last two decades, medical research has begun to make extensive use of products of human origin in therapeutics, oncology, and most recently, in genetic diseases. This has raised many ethical issues involving patient rights, including issues of consent. Besides informed consent, researchers should address several topics when designing studies using human tissues. Reward for the patient should be kept minimal. Sample transfer should be organized along non-profit lines, at least in Europe. Sampling procedures should be at no risk for human volunteers, and at minimal risk for patients. Biosafety aspects should be addressed, in particular when international collaborations are intended or when collaboration is existing between academia and industry. Regulations on importation and exportation of human tissues should be observed. Data acquisition and storage should be addressed in accordance with national data protection regulations, in particular when using computerized databases. If follow-up information is to be taken, the authorization for such information should be requested. The right for patient?s information (or for no information) on the research results should also be addressed. The issues of validation and patenting should be also solved, usually by informing the patient that he/she will have no commercial rights on potential research results. The patient should be told if the samples are transferred to another research laboratory or private company. Samples and related data should be destroyed on request at any time point during the course of the study. If possible, traceability of the donor should be ensured.

Narrative summary: The article notes growing concerns regarding the use of human tissues in biobanks or biorepositories, especially related to ownership and profit. It notes that ethical and regulatory guidance is vague and varies widely between countries. There are no international guidelines. The article suggests that these should be based on bioethical principles and international human rights law. It then delves into specific distinguishing features of proteomics. Firstly, proteomics allows for the investigation of environmental effects over time – it is dynamic while genomics is static. “As a logical consequence, they often require repeated analyses over the course of disease. Of course, anonymization of probes is not directly compatible with such follow-up studies.“ It suggests that this problem “can be circumvented by contracting a third party for anonymization and follow-up tasks, so that the biological information never comes in to contact with the patient’s identity.” It then points to two further distinguishing features: the need for large quantities of blood in proteomics studies, and its inability to enhance genomic information. Although these features remain relevant, the situation is reversed in 2020: very small quantities of blood are required, and much enhancement of genomic information is possible. The paper then suggests a series of recommendations essentially based on medical ethics codes. Of these, the most novel and important relates to the issue of intellectual property, proteomics, and the sharing of benefits. Finally, the author stressed the importance of informed consent with several options for levels of data sharing and privacy risks.

Reviewer: Sebastian Porsdam Mann, Peter Vilmos Treit

Themes
- Sensitive data/discrimination
  - Discrimination
  - Sensitivity of data/personal data
- Identifiability/privacy
  - Privacy
  - Identification/identifiability
- Incidental findings/reuse
  - Individual relevance of results
- Standards and quality control
  - Public databases: access and data integrity
  - Need for standards
- Conflicting rights and duties
  - Tensions between duties to patients and to science
  - Anonymization and data linkage
  - Economic incentives for data sharing/undue inducement
  - Public v Private (profit)
  - Patient/data subject rights
  - Intellectual property
- Need for normative international guidelines
  - Lack of normative guidelines
  - International collaboration
- Aims and goals of clinical proteomics
  - Need for positive ethics beyond regulations
  - Need for discussion within field
- Benefits/Justice
  - Profit/benefit sharing
  - Data sharing
- Regulation
  - Informed Consent

Reviewer: Sebastian Porsdam Mann, Peter Vilmos Treit
Issues
- Privacy
- Identification/identifiability
- Profit/benefit sharing
- Data sharing
- Lack of international ethical framework/guidance
- Economic incentives for data sharing
- Private v Public (Profit, ownership)
- Regulation
- Informed consent
- Rights
- Intellectual Property
- Need for standards
- International collaboration

ID 48757355: Twyman (2012)

Twyman R M and Chadwick Ruth. 2012. Proteomics. In , edited by , 642-649. San Diego: Academic Press.
Abstract: Human samples and large derivative data sets are necessary for biomedical proteomics research and the development of new drugs using proteomics data. The likelihood that such data will be used in a predictive manner raises ethical concerns,

principally the impact on privacy, which means it is necessary to balance the needs of medical research with the needs and rights of the patient. Another important ethical challenge is the issue of property rights (and intellectual property rights) arising from the collection, storage, and dissemination of biological samples and the proteomic data generated from them. There is no consistent international regulatory framework in place. The outstanding ethical issues are currently addressed by seeking the informed consent of the donor, but it is not clear that current forms of informed consent provide donors and patients with sufficient information and suitable choices.

Narrative summary: This was the first article we found that distinguished clearly between genomics and proteomics and focused on the ethical issues specific to proteomics. The author notes that "Proteomics provides a much more robust and representative picture of the functioning cell than do other forms of large-scale biology, such as genome sequencing or the global analysis of gene expression; therefore, the potential ethical risks associated with sample and data misuse are greater." He notes the importance of identifiability: "[t]he ethical issues raised by the separation of proteins reflect the fact that such methods could, and indeed do, provide molecular fingerprints that can be used to identify individuals, ethnic groups, and, in a clinical setting, groups of individuals with or susceptible to specific diseases," and later makes clear the link between identification and the possibility "that protein fingerprints could be used to discriminate against individuals and groups... Where such data are used solely for the clinical benefit of the patient, this would be acceptable. The danger lies in any possibility that proteomic analysis would be used to predict complex medical or even psychological outcomes and deny individuals with specific proteomic profiles insurance or employment or cause invasions of privacy."
The author notes that these issues are similar to their genomic counterparts, but that the proteomics context renders them "more imperative ethical challenges compared to geno¬mics, with the greater imperative resulting from the greater resolution, diversity, and dynamism of proteomics data compared to DNA sequences, and the wider range of samples that can be used for proteomic analysis compared to DNA. For example, proteomics can be applied to ancient samples in which DNA has degraded beyond use, to fixed tissue specimens, and to body tissues and fluids that lack DNA, such as serum, red blood cells, and spinal fluid. This means that proteomics may provide precise molecular data in cases in which DNA evidence is impossible to secure." He also points to another significant difference: "DNA resources are potentially infinite, whereas proteomic resources are not…However, any data derived from the samples can be used repetitively, either for comparison with a different data set or, where the raw data are stored, for mining to address any number of different questions." He therefore underscores the calls for broader or more dynamic consent options, giving participants more information and further options at the point of consent to reflect the greater possibilities of reuse and the lesser foreseeability of these uses. The article also calls for high levels of data security and anonymization wherever possible.
Most importantly, the article notes that "[t]he regulatory oversight for the use of human material for proteomic research differs in different countries and is evolving, but harmonization is being developed in the form of the Universal Declaration on the Human Genome and Human Rights proposed by the United Nations in 1998, which focuses on protection of human health and safety, human (especially patient) rights, commercial rights, intellectual property rights, and international regulations…..Another important ethical challenge is the issue of property rights (and intellectual property rights) arising from the collection, storage, and dissemination of biological samples and the proteomic data generated from them. A harmonized international regulatory framework would be beneficial in this context, but no consistent framework is in place. The outstanding ethical issues are currently addressed by seeking the informed consent of the donor, but it is imperative that informed consent provides donors and patients with sufficient information and suitable choices."

Reviewer: Sebastian Porsdam Mann, Peter Vilmos Treit
Themes
- Sensitive data/discrimination
  - Discrimination
  - Sensitivity of data/personal data
- Identifiability/privacy
  - Privacy
  - Identification/identifiability
  - Forensic use
  - Third party identifiability
- Incidental findings/reuse
  - Incidental findings/Secondary analysis
  - Uncertainty
  - Individual relevance of results
- Standards and quality control
  - Public databases: access and data integrity
  - Need for standards
  - Interoperability/inefficiencies
- Conflicting rights and duties
  - Tensions between duties to patients and to science
  - Anonymization and data linkage
  - Public v Private (profit)
  - Patient/data subject rights
  - Intellectual property
- Need for normative international guidelines
  - Lack of normative guidelines
  - International collaboration
  - Public awareness/public opinion
- Aims and goals of clinical proteomics
  - Need for positive ethics beyond regulations
  - Need for discussion within field
- Benefits/Justice
  - Data sharing
- Regulation
  - Informed Consent

Reviewer: Sebastian Porsdam Mann , Peter Vilmos Treit
Issues
- Discrimination
- Privacy
- Identification/identifiability
- Data sharing
- Sensitivity of data/personal data
- Public databases: access and data integrity
- Lack of international ethical framework/guidance
- Private v Public (Profit, ownership)
- Informed consent
- Rights
- database interoperability/inefficiencies
- Third party identifiability

*Grouping tables*

The following tables were also outputted by the Eppirreviewer software tool. They contain codes (issues) grouped into themes.

*Theme 1: Identifiability/privacy*

| Code (Issue) | Count | Studies |
|---|---|---|
| Privacy | 10 | Reymond(2003)<br>Nestler (2004)<br>Twyman (2012)<br>Gupta (2014)<br>Laatsch (2014)<br>Li (2016)<br>Parker (2016)<br>Martens (2017)<br>Boonen (2019)<br>Critselis (2019) |
| Identification/identifiability | 10 | Reymond(2003)<br>Nestler (2004)<br>Twyman (2012)<br>Gupta (2014)<br>Laatsch (2014) |

| | | |
|---|---|---|
| | | Li (2016)<br>Parker (2016)<br>Martens (2017)<br>Boonen (2019)<br>Critselis (2019) |
| Forensic use | 5 | Twyman(2012)<br>Laatsch (2014)<br>Li (2016)<br>Parker (2016)<br>Boonen (2019) |
| Third party identifiability | 2 | Beck (2004)<br>Twyman(2012) |

*Sensitive data/discrimination*

| Code | Count | Studies |
|---|---|---|

| | | |
|---|---|---|
| Discrimination | 9 | Reymond(2003)<br>Nestler (2004)<br>Twyman (2012)<br>Gupta (2014)<br>Laatsch (2014)<br>Li (2016)<br>Parker (2016)<br>Boonen (2019)<br>Critselis (2019) |
| Sensitive data | 9 | Reymond(2003)<br>Nestler (2004)<br>Twyman (2012)<br>Gupta (2014)<br>Laatsch (2014)<br>Li (2016)<br>Parker (2016)<br>Boonen (2019)<br>Critselis (2019) |

*Incidental findings/reuse*

| Code | Count | Studies |
|---|---|---|
| Incidental findings/Secondary analysis | 5 | Nestler(2004)<br>Twyman(2012)<br>Gupta (2014)<br>Boonen (2019)<br>Critselis (2019) |
| Uncertainty | 4 | Twyman(2012)<br>Gupta (2014)<br>Boonen (2019)<br>Critselis (2019) |
| Individual relevance of results | 6 | Reymond(2003)<br>Beck (2004)<br>Nestler (2004)<br>Twyman (2012)<br>Gupta (2014) |

| | | |
|---|---|---|
| | | Boonen (2019) |

*Standards and quality control*

| Code | Count | Study |
|---|---|---|
| Public databases: access and | 9 | Reymond(2003)<br>Jackson (2010)<br>Twyman (2012)<br>Gupta (2014)<br>Laatsch (2014)<br>Li (2016)<br>Martens (2017)<br>Boonen (2019)<br>Critselis (2019) |

| | | |
|---|---|---|
| Need for standards | 10 | Reymond(2003)<br>Beck (2004)<br>Nestler (2004)<br>National (2006)<br>Jackson (2010)<br>Twyman (2012)<br>Gupta (2014)<br>Martens (2017)<br>Boonen (2019)<br>Critselis (2019) |
| Interoperability/inefficiencies | 6 | Jackson(2010)<br>Twyman(2012)<br>Gupta (2014)<br>Laatsch (2014)<br>Martens(2017)<br>Boonen (2019) |

| | | |
|---|---|---|
| | | |
| Sample Preparation / Storage | | |
| Data | | HUPO PSI |

*Conflicting rights and duties*

| Code | Count | Study |
|---|---|---|
| Tensions between duties to patients and to science | 8 | Reymond(2003)<br>Beck (2004)<br>Liska (2004)<br>Nestler (2004)<br>National (2006) |

| | | |
|---|---|---|
| | | Twyman (2012)<br>Boonen (2019)<br>Critselis (2019) |
| Anonymization and data linkage | 6 | Reymond(2003)<br>Beck (2004)<br>Twyman (2012)<br>Martens (2017)<br>Boonen (2019)<br>Critselis (2019) |
| Economic incentives for data sharing/undue inducement | 2 | Reymond (2003)<br>Boonen (2019) |
| Public v Private (profit) | 8 | Reymond(2003)<br>Liska (2004)<br>National (2006)<br>Twyman (2012)<br>Gupta (2014)<br>Özdemir (2015) |

| | | |
|---|---|---|
| | | Boonen (2019)<br>Critselis (2019) |
| Patient/data subject rights | 8 | Reymond(2003)<br>Liska (2004)<br>Nestler (2004)<br>National (2006)<br>Twyman (2012)<br>Gupta (2014)<br>Boonen (2019)<br>Critselis (2019) |
| Intellectual Property | 7 | Reymond(2003)<br>Nestler (2004)<br>National (2006)<br>Twyman (2012)<br>Gupta (2014)<br>Boonen (2019) |

| | | |
|---|---|---|
| | | Critselis (2019) |

*Need for normative international guidelines*

| Code | Count | Study |
|---|---|---|
| Lack of normative guidance | 11 | Reymond(2003)<br>Beck (2004)<br>Liska (2004)<br>Nestler (2004)<br>National (2006)<br>Jackson (2010)<br>Twyman (2012)<br>Gupta (2014)<br>Özdemir (2015)<br>Boonen (2019)<br>Critselis (2019) |

| | | |
|---|---|---|
| Trust | 6 | Liska (2004)<br><br>Nestler(2004)<br><br>Gupta (2014)<br><br>Özdemir(2015)<br><br>Holmes (2016)<br><br>Boonen (2019) |
| International collaboration | 10 | Reymond(2003)<br><br>Liska (2004)<br><br>Nestler (2004)<br><br>National (2006)<br><br>Jackson (2010)<br><br>Twyman (2012)<br><br>Gupta (2014)<br><br>Özdemir (2015)<br><br>Boonen (2019)<br><br>Critselis (2019) |

| | | |
|---|---|---|
| Public awareness/public opinion | 5 | Liska (2004)<br>Twyman(2012)<br>Gupta (2014)<br>Özdemir(2015)<br>Holmes (2016) |
| Social consequences | 4 | Liska (2004)<br>Nestler(2004)<br>Gupta (2014)<br>Özdemir(2015) |

*Aims and goals of clinical proteomics*

| Code | Count | Study |
|---|---|---|
| Funding priorities | 2 | Liska (2004)<br>Gupta (2014) |
| Aims of research | 4 | Beck (2003)<br>Liska (2004)<br>Gupta (2014)<br>Özdemir (2015) |

| | | |
|---|---|---|
| Need for positive ethics beyond regulation | 8 | Reymond(2003)<br>Liska (2004)<br>Nestler (2004)<br>Twyman (2012)<br>Gupta (2014)<br>Özdemir (2015)<br>Boonen (2019)<br>Critselis (2019) |
| Need for discussion within field | 7 | Reymond(2003)<br>Liska (2004)<br>Twyman (2012)<br>Gupta (2014)<br>Özdemir (2015)<br>Boonen (2019)<br>Critselis (2019) |

*Benefits/Justice*

| Code | Count | Study |
|---|---|---|

| | | |
|---|---|---|
| Profit/benefit sharing | 8 | Reymond(2003)<br>Liska (2004)<br>Nestler (2004)<br>National (2006)<br>Gupta (2014)<br>Özdemir (2015)<br>Boonen (2019)<br>Critselis (2019) |
| Data sharing | 7 | Reymond(2003)<br>Liska (2004)<br>Nestler (2004)<br>Twyman (2012)<br>Gupta (2014)<br>Boonen (2019)<br>Critselis (2019) |
| Access for low-and-middle-income countries | 3 | Liska (2004)<br>Gupta (2014)<br>Özdemir (2015) |
| Databases not globally representative | 1 | Gupta (2014) |

| | | |
|---|---|---|
| Solidarity | 4 | Liska (2004)<br>Nestler (2004)<br>Gupta (2014)<br>Özdemir (2015) |
| Altruism | 4 | Liska (2004)<br>Nestler (2004)<br>Gupta (2014)<br>Özdemir (2015) |
| Fairness | 4 | Liska (2004)<br>Nestler (2004)<br>Gupta (2014)<br>Özdemir (2015) |
| Authority/power | 2 | Gupta (2014)<br>Özdemir (2015) |

*Regulation*

| Code | Count | Study |
|---|---|---|
| GDPR | 2 | Boonen (2019)<br>Critselis (2019) |
| Informed Consent | 7 | Reymond(2003)<br>Beck (2004)<br>Nestler (2004)<br>Twyman (2012)<br>Gupta (2014)<br>Boonen (2019)<br>Critselis (2019) |

| | | |
|---|---|---|
| Other regulation | 9 | Liska (2004)<br>National(2006)<br>Jackson (2010)<br>Laatsch (2014)<br>Özdemir(2015)<br>Holmes (2016)<br>Li (2016)<br>Parker (2016)<br>Martens(2017) |

*Integration of new technologies and related fields*

| Code | Count | Study |
|---|---|---|
| Evolution of technology | 5 | Gupta (2014)<br>Özdemir(2015)<br>Martens(2017)<br>Boonen (2019)<br>Critselis (2019) |

| | | |
|---|---|---|
| Complicating factors | 5 | Gupta (2014)<br>Özdemir(2015)<br>Martens(2017)<br>Boonen (2019)<br>Critselis (2019) |
| Integration/other | 11 | Reymond(2003)<br>Beck (2004)<br>Liska (2004)<br>Nestler (2004)<br>National (2006)<br>Jackson (2010)<br>Twyman (2012)<br>Laatsch (2014)<br>Holmes (2016)<br>Li (2016)<br>Parker (2016) |
